# Evaluation of Virtual Screening Strategies for the Identification of γ-Secretase Inhibitors and Modulators

**DOI:** 10.3390/molecules27010176

**Published:** 2021-12-28

**Authors:** Alicia Ioppolo, Melissa Eccles, David Groth, Giuseppe Verdile, Mark Agostino

**Affiliations:** 1 Curtin Health and Innovation Research Institute, Curtin Medical School, Curtin University, Bentley, WA 6102, Australia; alicia.ioppolo@postgrad.curtin.edu.au (A.I.); melissa.eccles@postgrad.curtin.edu.au (M.E.); D.Groth@curtin.edu.au (D.G.); giuseppe.verdile@curtin.edu.au (G.V.); 2 School of Medical and Health Sciences, Edith Cowan University, Joondalup, WA 6027, Australia; 3 Curtin Institute for Computation, Curtin University, Bentley, WA 6102, Australia

**Keywords:** γ-secretase, virtual screening, molecular docking, pharmacophore model, Alzheimer’s disease

## Abstract

γ-Secretase is an intramembrane aspartyl protease that is important in regulating normal cell physiology via cleavage of over 100 transmembrane proteins, including Amyloid Precursor Protein (APP) and Notch family receptors. However, aberrant proteolysis of substrates has implications in the progression of disease pathologies, including Alzheimer’s disease (AD), cancers, and skin disorders. While several γ-secretase inhibitors have been identified, there has been toxicity observed in clinical trials associated with non-selective enzyme inhibition. To address this, γ-secretase modulators have been identified and pursued as more selective agents. Recent structural evidence has provided an insight into how γ-secretase inhibitors and modulators are recognized by γ-secretase, providing a platform for rational drug design targeting this protease. In this study, docking- and pharmacophore-based screening approaches were evaluated for their ability to identify, from libraries of known inhibitors and modulators with decoys with similar physicochemical properties, γ-secretase inhibitors and modulators. Using these libraries, we defined strategies for identifying both γ-secretase inhibitors and modulators incorporating an initial pharmacophore-based screen followed by a docking-based screen, with each strategy employing distinct γ-secretase structures. Furthermore, known γ-secretase inhibitors and modulators were able to be identified from an external set of bioactive molecules following application of the derived screening strategies. The approaches described herein will inform the discovery of novel small molecules targeting γ-secretase.

## 1. Introduction

γ-Secretase is an intramembrane aspartyl protease that performs regulated intramembrane proteolysis of Type-I transmembrane (TM) proteins [1], processing over 100 functionally diverse substrates [2,3]. It is a heterotetrametric enzyme complex [4] composed of presenilin (PS, of which there are two homologues, PS1 and PS2) [5], nicastrin (Nct) [6], anterior pharynx defective-1 (Aph-1) [7], and presenilin enhancer-2 (Pen-2) [8]. The most notable substrates of γ-secretase are Amyloid Precursor Protein (APP) [9] and Notch family receptors 1–4 [10]. Due to its role in APP processing, γ-secretase has been investigated for the development of disease-modifying therapeutics for Alzheimer’s Disease (AD), amongst other γ-secretase related disorders [11]. These molecules are classified as γ-secretase inhibitors (GSIs), which target the enzyme active site, and γ-secretase modulators (GSMs) that bind to an allosteric site [12,13].

Numerous GSIs, including avagacestat and semagacestat [14,15], have progressed to clinical trials; however, they have shown off-target effects, including worsening in cognition, the development of cancers, subcutaneous tissue disorders, and gastrointestinal problems [15,16]. These associated complications are believed to be mediated by GSIs broadly inhibiting, and thereby precluding, the release of biologically important intracellular domains (ICDs) [17]. Many first generation GSIs, including L-685,458, were peptidic transition state analogs that feature hydroxyethylene dipeptide isostere moieties, allowing them to bind to γ-secretase in a way that mimics the α-helical nature of the APP TM domain [18]. However, these molecules lack Notch-sparing activity and thus have not been pursued for development as therapeutic agents [19]. The identification of a region on the γ-secretase surface which is responsible for initial substrate binding [20] led to a focus on identifying GSIs that target the substrate-docking site of the enzyme. DAPT (N-[N-(3,5-diflurophenacetyl)-1-alanyl]-S-phenylglycine *t*-butyl ester), which binds at a site that is close to the enzyme catalytic site, but remains pharmacologically distinct, was the first orally active GSI [21]. Continued optimization of this GSI class led to the development of semagacestat (a carboxamide) and avagacestat (an arylsulphonamide). These GSIs have been assessed in clinical trials against AD, showing a dose-dependent decrease in the production of Aβ_40_ and Aβ_42_ in vitro and in vivo [14,15,22]; however, participants receiving various dosages of treatments showed a higher occurrence of health complications than the placebo group and, hence, were discontinued [15,16].

In recognition of problems related to the broad inhibition of cleavage, an alternative approach was proposed to identify molecules that selectively inhibit (i.e., modulate) the production of longer neurotoxic Aβ species while maintaining ICD generation from all substrates. Non-steroidal anti-inflammatory drugs (NSAIDs) were the first molecules seen to modulate Aβ levels [23,24], such that there is a decrease in Aβ_42_ production alongside a simultaneous increase in the production of the shorter, less pathogenic Aβ_38_ [25]. Numerous GSMs have since been identified with this type of activity toward γ-secretase [12,26]. Neurogenetics developed the first non-NSAID GSMs, which were based around aryl- or heteroarylimidazoles and anilinothiazoles and led to the development of NGP555 [26]. This molecule binds to an allosteric interface of γ-secretase between Pen-2, Nct, and the TM 3-4 loop of PS1-NTF [27]. In vitro activity assessment by ELISA has shown NGP555 to afford a dose-dependent decrease in Aβ_42_ and Aβ_40_ production, while increasing the production of Aβ_38_, and having no significant change to the Aβ total or the generation of ICDs from other substrates [26]. This GSM has shown no associated toxicity in animal studies and has since progressed to Phase I clinical trials [27]. Analogs of the NGP555 scaffold have been identified and generally afford increased structural rigidity and metabolic stability through structural replacement and rearrangements [26,28]. However, these derivatives are similar in structure to NGP555, and there has been limited progression in the identification of new GSM molecular scaffolds.

**Figure 1:**
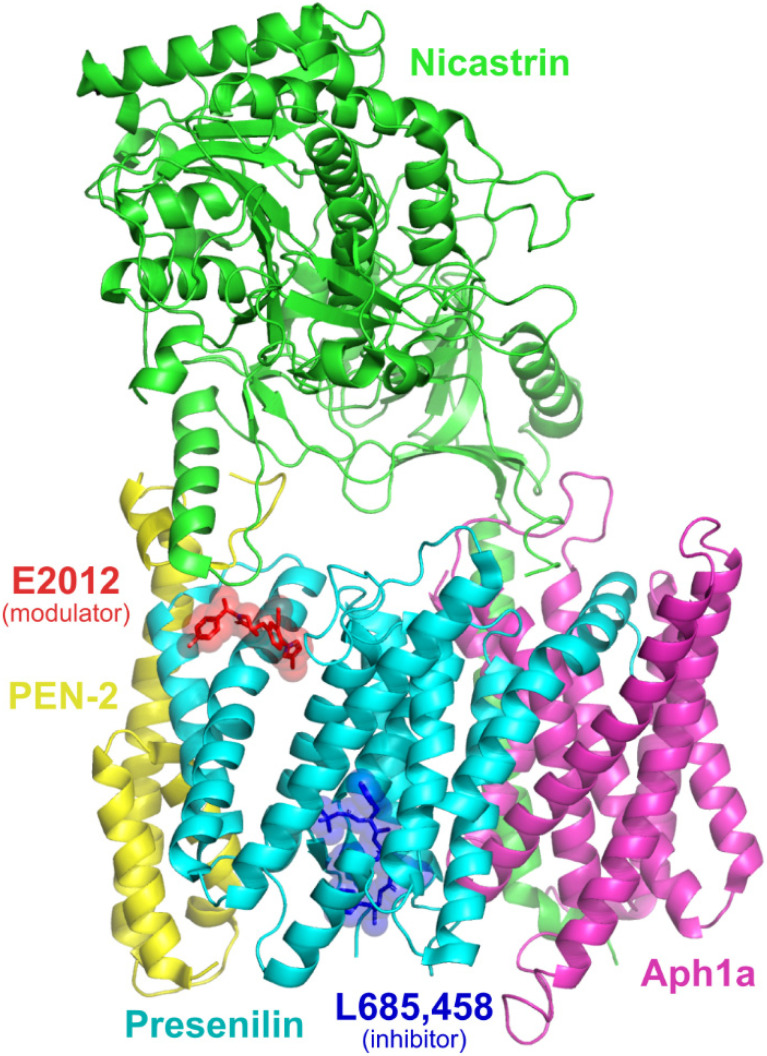
Co-complex of γ-secretase, L685,458, and E2012 (PDB 7D8X), illustrating the locations of the inhibitor and modulator sites.

As a multi-subunit TM protein, γ-secretase has eluded structural characterization until recently. Bai et al. characterized a series of γ-secretase *apo*-state ensembles (PDB 5FN3, 5FN4, 5FN5) [29] by cryoelectron microscopy, alongside a complex with the GSI DAPT (PDB 5FN2, although the DAPT molecule could not be atomically resolved [29]). Of note, among these structures is the presence of a density in two *apo*-state ensembles (PDB 5FN3, PDB 5FN4) that could not be ascribed to γ-secretase, and is hypothesized to be unidentified co-purified substrates [29]. Compounding this, TM2, a region believed to be important for substrate binding, is missing from several of these structures (PDB 5A63, PDB 5FN4, PDB 5FN5) [29,30]. More recently, atomic structures of γ-secretase enzymes in a complex with APP (PDB 6IYC [31]) and Notch (PDB 6IDF [32]) substrates have been determined. These structures reveal the formation of an antiparallel three-stranded β-sheet between the C-terminal portion of substrates, with two induced β-strands, β1 (residues 287–290 when bound with APP) and β2 (377–381) from TM6 and TM7 of PS1. This structural rearrangement was not evident in the DAPT-bound γ-secretase [29] and thus is predicted to be important in substrate proteolysis and/or recognition [31,32]. The most recent structures of γ-secretase feature bound inhibitors (semagacestat, avagacestat, L685,458) and a bound modulator (E2012) (Figure 1), providing a basis to pursue structure-based drug design targeting γ-secretase [33].

Due to the historic lack of high-resolution γ-secretase structures, the design of GSIs and GSMs has been largely focused on high throughput screening and medicinal chemistry efforts to modify existing lead molecules, resulting in limited chemical diversity. The availability of high-resolution γ-secretase structures in complex with APP, Notch, and, most recently, GSIs and GSMs, provides the opportunity to use structure-based approaches for the identification of novel molecules that may have inhibitory or modulatory activity at γ-secretase. In this study, docking and pharmacophore modelling approaches were explored for their ability to reproduce the structures of inhibitors and modulators structurally co-complexed with γ-secretase, and virtual screening protocols for identifying both γ-secretase inhibitors and modulators based on these approaches were developed and validated.

## 2. Results

### 2.1. Evaluation of Docking and Pharmacophore Modelling for Pose Prediction of γ-Secretase Inhibitors and Modulators

**Figure 2:**
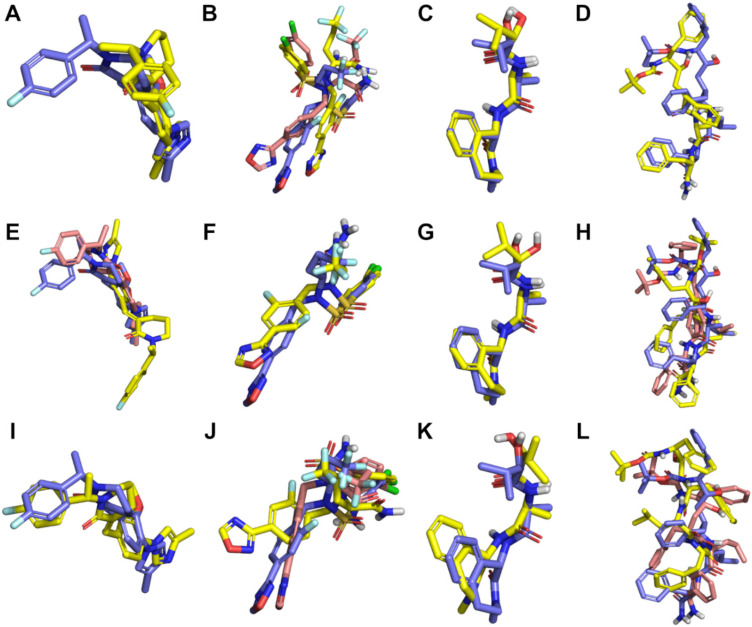
Best poses obtained by Glide HTVS docking (**A**–**D**), Glide SP docking (**E**–**H**), and ePharmacophore fitting (**I**–**L**) for E2012 (**A**,**E**,**I**), avagacestat (**B**,**F**,**J**), semagacestat (**C**,**G**,**K**), and L685,458 (**D**,**H**,**L**). In all panels, the structure depicted with blue-violet carbons is the ligand structure from its co-complex with γ-secretase; the structure depicted with yellow carbons is the best fitting, top-ranked pose obtained for the ligand by the given method, and the structure depicted with pink carbons (if present) is the best fitting pose—regardless of rank—obtained for the ligand by the given method. (**A**)**.** E2012 docked by Glide HTVS to PDB 7D8X (RMSD = 3.5 Å). (**B**)**.** Avagacestat docked by Glide HTVS to PDB 6IDF (RMSD = 6.1 Å) and to PDB 6LQG (RMSD = 5.2 Å for 2nd ranked pose). (**C**)**.** Semagacestat docked by Glide HTVS to PDB 6LR4 (RMSD = 0.6 Å). (**D**)**.** L685,458 docked by Glide HTVS to 6IYC (RMSD = 2.9 Å). (**E**)**.** E2012 docked by Glide SP to PDB 6LQG (RMSD = 7.8 Å) and PDB 6IYC (RMSD = 2.2 Å for 22nd ranked pose). (**F**)**.** Avagacestat docked by Glide SP to PDB 6LQG (RMSD = 1.9 Å). (**G**)**.** Semagacestat docked by Glide SP to PDB 6LR4 (RMSD = 1.0 Å and 0.8 Å for 3rd ranked pose). (**H**)**.** L685,458 docked by Glide SP to PDB 7D8X (RMSD = 4.0 Å) and PDB 6IYC (RMSD = 2.3 Å for 12th ranked pose). (**I**)**.** E2012 fitted to ePharmacophore derived from PDB 7D8X (RMSD = 1.9 Å). (**J**)**.** Avagacestat fitted to ePharmacophore derived from PDB 6LQG (RMSD = 3.1 Å and 2.9 Å for 3rd ranked pose). (**K**)**.** Semagacestat fitted to ePharmacophore derived from PDB 7C9I (RMSD = 1.5 Å). (**L**)**.** L685,458 fitted to ePharmacophore derived from PDB 7D8X (RMSD = 5.1 Å) and PDB 6IYC (RMSD = 4.1 Å).

Docking and pharmacophore modelling were initially evaluated for their ability to reproduce the bound states of γ-secretase inhibitors and modulators. Evaluation was conducted both against the cognate structure/pharmacophore, as well as against other structures/pharmacophores (Figure 2; Supplementary Materials Tables S1–S3). Glide HTVS (Figure 2A–D; Supplementary Materials Table S1) generally achieved poorly fitting poses, the only exception being for the semagacestat docked to the cognate structure (PDB 6LR4). Glide SP (Figure 2E–H; Supplementary Materials Table S2) generated well-fitting poses of E2012 against the cognate structure (PDB 7D8X), PDB 6LGQ, and PDB 6IYC; however, these poses were not highly ranked by GlideScore. Glide SP generated well-fitting poses of avagacestat against the cognate structure (PDB 6LGQ) and both structures of γ-secretase representing substrate-bound conformations (PDB 6IYC and PDB 6IDF), and, in all circumstances, these were the top-ranked poses. Glide SP generated well-fitting poses of semagacestat against all structures, although, in some circumstances (PDB 6IYC and 6LGQ), slightly better fitting poses are found lower down the ranked list (but nonetheless, still within the three best-ranked poses). Glide SP generated well-fitting poses of L685,458 only against the structure of γ-secretase representing the APP-bound conformation (PDB 6IYC), however, these are not highly ranked by GlideScore, and docking to either cognate structure (PDB 7C9I and PDB 7D8X) failed to produce well-fitting poses. While pose prediction via ePharmacophore models generally performed poorly (Figure 2I–L; Supplementary Materials Table S3), a noteworthy exception was seen for E2012 against the cognate structure (PDB 7D8X), where a well-fitting pose was achieved and ranked at the top of the list by PhaseScreenScore. Taken altogether, these results suggest the ability of Glide SP to reproduce the bound structures of traditionally drug-like γ-secretase inhibitors, while the ePharmacophore approach is preferred for predicting the bound structures of γ-secretase modulators.

### 2.2. Derivation of Virtual Screening Strategies for γ-Secretase Inhibitors and Modulators

As Glide HTVS and ePharmacophore screening are relatively fast approaches, it is preferable to perform an initial screen of a large library by either of these methods, select highly ranked molecules, then screen the selected molecules with a slower/more accurate approach, such as Glide SP or more thorough approaches to determining binding free energy [34,35]. Thus, optimal virtual screening approaches for γ-secretase inhibitors and modulators were investigated and designed with this in mind.

The ability of Glide HTVS and ePharmacophore screening to identify known inhibitors and modulators of γ-secretase from decoys generated by DUD-E was initially investigated (Table 1, Supplementary Materials Figures S1–S4). The best performing structure–method combinations for identifying γ-secretase inhibitors were to use Glide HTVS against the APP-bound conformation of γ-secretase (PDB 6IYC; AUC = 0.67, optimal MCC = 0.16 for top 7% of screen) and to screen against the ePharmacophore derived from the L685,458-bound conformation of γ-secretase (PDB 7C9I; AUC = 0.63, optimal MCC = 0.17 for top 6% of screen); the latter approach is preferred as it achieves a slightly higher MCC within a slightly smaller range of the screen. When screening for γ-secretase modulators, Glide HTVS fares poorly regardless of the structure employed, with optimal MCCs in all cases determined to be less than 0.1, suggesting near-random performance for this approach. In selected cases—specifically, PDBs 6IYC and 7D8X (γ-secretase in complex with L685,438 and the modulator E2012)—optimal MCCs above 0.1 are obtained by ePharmacophore screening, with the best performance achieved using the 7D8X-derived ePharmacophore (AUC = 0.73, MCC = 0.16 for top 7% of screen). Thus, in the context of screening for both γ-secretase inhibitors and γ-secretase modulators, the ePharmacophore approach was preferred for the initial stage of screening, although with different structures being preferred for each ligand class.

The respective sets of top ranked molecules following screening by the ePharmacophore approach were then screened by Glide SP at each of the γ-secretase structures (Table 2, Supplementary Materials Figures S5–S8). Noting that the correct poses for E2012 could only be accurately generated and ranked by screening against the 7D8X-derived ePharmacophore (Figure 1; Supplementary Materials Table S3), it was decided to examine whether allowing Glide SP to sample ligands in a fully flexible fashion (as is the default) or whether only to refine ePharmacophore-derived poses led to an improved enrichment of actives. When full ligand flexibility is allowed during rescreening by Glide SP, the APP-bound conformation of γ-secretase (PDB 6IYC) gives near perfect enrichment to the remaining γ-secretase inhibitors (AUC = 0.99, MCC = 0.93 for top 11% of screen). With the exception of the avagacestat-bound conformation (PDB 6LQG), the remaining structures all perform excellently for enriching the remaining γ-secretase inhibitors, achieving AUCs between 0.74 and 0.97, and peak MCCs between 0.65 and 0.83. In comparison, refining the initial ePharmacophore-derived poses achieves poorer, although still excellent, results for enriching γ-secretase inhibitors from the set of top ranked molecules remaining following screening against the 7C9I-derived ePharmacophore; notably, vastly improved results are seen for screening against PDB 6LQG. In contrast to enriching γ-secretase inhibitors, the best strategy for enriching γ-secretase modulators appears to be to refine by Glide SP (i.e., not to incorporate flexible ligand sampling) the initial ePharmacophore-derived poses against PDB 7D8X (AUC = 0.70, MCC = 0.39 for top 9% of screen), which achieves a modest improvement over the best performing structure when full ligand flexibility is incorporated (for PDB 7C9I; AUC = 0.64, MCC = 0.26 for top 10% of screen).

The poses of the top ranked ligands obtained from the optimal Glide SP re-screens for γ-secretase inhibitors and γ-secretase modulators were then further rescored by Prime MMGBSA. This rescoring gives further enrichment of γ-secretase modulators, but fails to further enrich γ-secretase inhibitors (Table 3, Supplementary Materials Figure S9).

**Figure 3:**
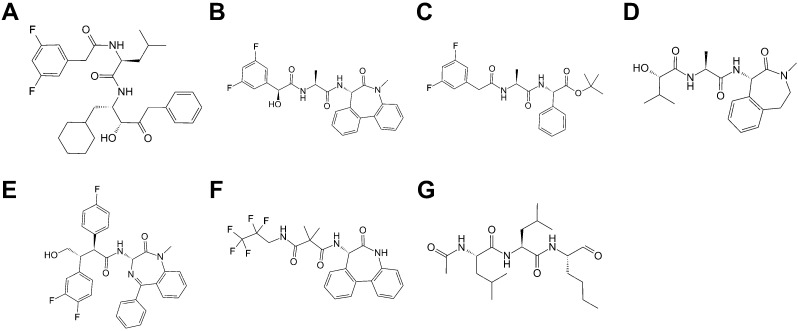
γ-secretase inhibitors identified from the validation library following application of the optimized virtual screening strategy. Aβ_40_ IC50 values listed as reported in [45]: (**A**). Bristol Myers Squibb hydroxyethylene active site-binding inhibitor (160 nM). (**B**). LY411575 (30 pM). (**C**). DAPT (20 nM). (**D**). Semagacestat (15 nM). (**E**). (2*S*-3*R*)-3-(3,4-difluorophenyl)-2-(4-fluorophenyl)-4-hydroxy-N-((3*S*)-2-oxo-5-phenyl-2,3-dihydro-1*H*-benzo[e][1,4]diazepin-3-yl)butyramide (60 pM). (**F**). RG-4733 (14 nM). (**G**). ALLN (50 μM).

**Figure 4:**
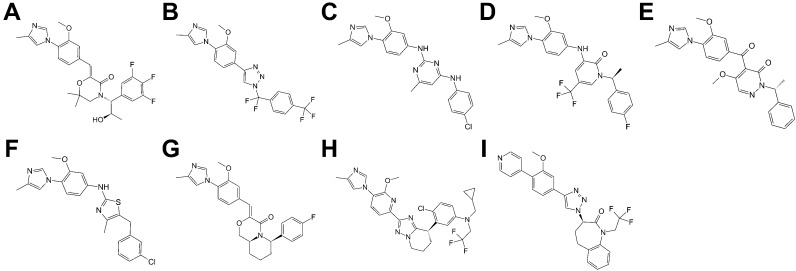
γ-secretase modulators identified from the validation library following application of the optimised virtual screening strategy. Aβ_42_ IC50 values listed as reported in [28]: (**A**). Eisai morpholinone (5 nM). (**B**). Merck 1,2,3-triazole (2.245 nM). (**C**). Roche pyrimidine (70 nM). (**D**). Merck aminopyridone (101 nM). (**E**). Schering keto-linked modulator (2.5 μM). (**F**). Roche thiazole (210 nM). (**G**). Eisai bicyclic morpholinone (201 nM). (**H**). Eisai bicyclic triazole (6 nM). (**I**). Merck 1,2,3-triazole with bicyclic substituent (80 nM).

**Figure 5:**
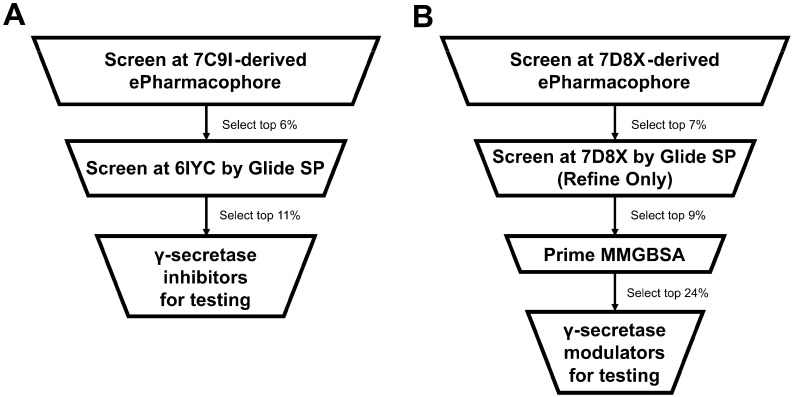
Overview of the γ-secretase inhibitor (**A**) and γ-secretase modulator (**B**) screening strategies.

Considering the results, the optimal strategy for screening for γ-secretase inhibitors was thus chosen as being to select the top 6% of molecules obtained from screening at the 7C9I-derived ePharmacophore, followed by selecting the top 11% of this subset of molecules rescreened at PDB 6IYC by Glide SP with full ligand flexibility with no further rescoring. The γ-secretase inhibitors remaining following the application of this strategy include DAPT and semagacestat, as well as molecules with related scaffolds (Figure 3). L685,458 (which features a peptide-like scaffold as per DAPT and semagacestat) and avagacestat (which features a scaffold unrelated to all of the other structurally co-complexed γ-secretase inhibitors) are not among the known inhibitors selected. The optimal strategy for screening for γ-secretase modulators was to select the top 7% of molecules obtained from screening at the 7D8X-derived ePharmacophore, followed by selecting the top 9% of this subset of molecules rescreened at PDB 7D8X by refining ePharmacophore-derived poses with Glide SP, followed by selecting the top 24% following Prime MMGBSA rescoring of the Glide SP-rescreened subset. The γ-secretase modulators remaining following the rescreening are derived from a diverse chemical series (Figure 4) [36,37,38,39,40,41,42,43,44]. The optimal virtual screening strategies are summarized in Figure 5.

### 2.3. Application of Optimal Virtual Screening Strategies to the ZINC15 Investigational Set

The ability of the optimal virtual screening strategies to identify known γ-secretase inhibitors and γ-secretase modulators was further investigated in the context of the ZINC15 Investigational set. This set includes several thousands of molecules with bioactivity at diverse targets, as well as a small selection of γ-secretase inhibitors and modulators (including avagacestat, semagacestat, and E2012). Thus, the set forms a reasonable external library for further validation.

The molecules identified from the ZINC15 Investigational set following application of the optimal virtual screening strategy for identifying γ-secretase inhibitors are listed in Table 4 (and displayed in Supplementary Materials Figure S10). Many of the molecules identified in the virtual screen are known inhibitors of other proteases. Although none of the structurally co-complexed γ-secretase inhibitors are identified in this screen, the γ-secretase inhibitor crenigacestat—structurally related to semagacestat—is among the molecules identified. With the exception of crenigacestat, it is believed that none of these molecules have been previously tested against γ-secretase, although molecules originally identified against some of the identified target classes have been demonstrated to have activity against γ-secretase, including protease inhibitors [46], kinase inhibitors [47,48], and transcription factors [49].

Shape similarity calculations between the docked solutions of the molecules listed in Table 4 and the docked conformations of avagacestat, semagacestat, and L685,458 to PDB 6IYC best fitting the respective co-complex structures (see Supplementary Materials Table S2; these structures representing suitably refined conformations of these molecules in the field of the receptor structure being used for the screen) reveal similarity between some of the selected molecules with known γ-secretase inhibitors (Supplementary Materials Table S4). Specifically, limited similarity between oxacillin and avagacestat was identified (similarity score = 0.200), and limited similarity between droxinavir and L685,458 was identified (similarity score = 0.200). Several molecules were identified to have limited to moderate similarity to semagacestat, specifically Foxy-5 (similarity score = 0.216), oxacillin (similarity score = 0.264), droxinavir (similarity score = 0.302), oprozomib (similarity score = 0.410), and the known γ-secretase inhibitor crenigacestat (similarity score = 0.605). Analysis of the interactions made by the molecules bearing at least limited similarity to known γ-secretase inhibitors (>0.200) with γ-secretase (Supplementary Materials Figure S11) reveal that the majority of these molecules bear polyamide functionality (or a related functionality, such as sulfonamide in the case of avagacestat, keto in the case of oprozomib, and carboxylate in the case of oxacillin) that forms hydrogen bonds with one or more of Lys380, Gly382, Leu432, and Ala434 of presenilin. Further notable interactions are made by oxacillin, which forms hydrogen bonds with the catalytic Asp385 of presenilin (modelled as neutral by PROPKA) and Foxy-5, which forms a salt bridge with Arg377 of presenilin.

The molecules identified from the ZINC15 Investigational set following application of the optimal virtual screening strategy for identifying γ-secretase modulators are listed in Table 5 (and displayed in Supplementary Materials Figure S12). As per the screen of this library for identifying γ-secretase inhibitors, several of the molecules identified by the screen are also known as inhibitors of proteases. The γ-secretase modulator E2012 is among the molecules identified by the screen. Shape similarity calculations (Supplementary Materials Table S5) between the docked solutions of the molecules listed in Table 5 and the top pose of E2012 derived from fitting to the 7D8X-derived ePharmacophore (see Supplementary Materials Table S3; this structure representing the E2012 pose is the most similar to the conformation in the co-complex structure) reveal some similarity between E2012 as placed following the screen (similarity score = 0.317) and ocinaplon (similarity score = 0.244). Interaction analysis (Supplementary Materials Figure S13) reveals that E2012 and ocinaplon both form π–π interactions with Phe177 of presenilin, while E2012 also forms hydrogen bonds with Tyr106 and Tyr240 of presenilin. In all cases, a substantial portion of the molecules extends out into space likely occupied by membrane lipids.

## 3. Discussion

The virtual screening performances for the optimal approaches largely reflect the pose prediction performance, reinforcing the importance of accurate structural predictions in achieving high performing virtual screening approaches. In particular, accurate structural predictions for γ-secretase modulators are only possible via the pharmacophore-based approach, with the best virtual screening performances being centered on the use of pharmacophore-derived poses and their subsequent refinement, rather than redocking with full ligand flexibility. The γ-secretase modulator site is unusual among drug-binding sites in that it is anticipated to be partly membrane-exposed. By comparison, the γ-secretase inhibitor site is largely enclosed within the protein, similar to the typically druggable sites of membrane proteins such as G protein-coupled receptors. Thus, the modulator site represents a type of site that is unlikely to be considered in the development of molecular docking and scoring approaches, which, in turn, may connote the use (and indeed, preference) of pharmacophore-derived poses and further refinements to these in screening for modulators. For γ-secretase inhibitors, there is a slight preference towards the use of pharmacophore-based virtual screening in the initial stage of screening, although docking generally achieves much better pose prediction performance. A challenge remains in accurately predicting the structures of peptide-like inhibitors, which may be overcome through using specialized peptide docking approaches [84,85,86], although there is evidently no difficulty in selecting such molecules by the screening approaches presented here. The virtual screening strategies presented here incorporate both ligand- and structure-based approaches, whereas these have been investigated individually in previous studies [87,88].

A further consideration of the derived strategies is that the γ-secretase structures used in this study are all bound to either drug-like ligands or substrates, which, in turn, will promote the selection of specific γ-secretase conformations and may bias certain ligand classes. In particular, where pharmacophore models are derived from considering a bound ligand, this may result in the exclusive identification of ligands similar to that against which the pharmacophore was generated. This, in turn, may account for the particular screening strategy derived for the identification of γ-secretase modulators, which centers on the use of the E2012-bound structure. While the chemotypes of γ-secretase modulators identified thus far largely center around molecules similar to E2012, the present strategy identifies molecules featuring diverse variations on this scaffold (Figure 4). We anticipate that by selecting ligands/poses satisfying a majority of pharmacophore points rather than all points (as done throughout this study), and by incorporating structures bound to different ligands/substrates (as performed for γ-secretase inhibitors), possible bias can be reduced.

The application of the derived virtual screening approaches to the ZINC15 Investigational set suggests the possible general utility of the approaches. The majority of top-ranking molecules selected from the ZINC15 Investigational set in the context of screening for either γ-secretase inhibitors or γ-secretase modulators target proteases, illustrating the ability of the approaches to identify molecules with similar pharmacological functions, and the possibility of a conserved chemotype among these. Indeed, molecules previously identified as protease inhibitors at other proteins are substantially enriched by the screening approaches relative to their presence in the ZINC15 Investigational set (Supplementary Materials Figure S14). In addition to protease inhibitors, a substantial proportion of top-ranking molecules identified from the ZINC15 investigational set in the application of the γ-secretase inhibitor screening strategy target G protein-coupled receptors. While this could be a consequence of the frequent investigation of GPCRs as drug targets, it is notable, as the drug-binding sites of most GPCRs and the inhibitor site of γ-secretase are similar in that they both occur in protein cavities within the membrane, suggesting a degree of physicochemical similarity between sites and, hence, the selection by the screening approach of GPCR-targeting molecules. While the set contains a variety of γ-secretase inhibitors, including all of those co-complexed with the γ-secretase structures utilized in this study, only crenigacestat, an inhibitor under investigation for its activity against Notch [89], was identified by the virtual screen. This, in turn, suggests a degree of possible bias for the selection of particular chemotypes in the virtual screening strategy, which is anticipated to be able to be addressed as new structures of γ-secretase bound to different ligands and different substrates are solved.

In conclusion, we have developed multi-staged virtual screening strategies for predicting γ-secretase inhibitors and modulators. We have explored the application of docking- and pharmacophore-based approaches for both accuracy of pose prediction and screening for identifying both γ-secretase inhibitors and modulators. The derived strategies are anticipated to inform the discovery of new molecules targeting γ-secretase.

## 4. Materials and Methods

### 4.1. Selection and Preparation of γ-Secretase Structures

Unless otherwise noted, all calculations in this study were performed using tools from Schrodinger Suite 2019-4 (Schrodinger LLC, New York, NY, USA). Structures of γ-secretase were obtained from the Protein Data Bank and are detailed in Supplementary Materials Table S6. Structures were prepared using the Protein Preparation Wizard within Maestro 12.2. Missing side chains and loops (exempting the very large intracellular loop of presenilin and the presenilin N-terminal domain) were added using Prime, and protonation states for titratable residues were determined using PROPKA [90]. Structures were subject to Impref minimization, constraining atomic coordinates to a root-mean-squared deviation (RMSD) of 0.3 Å from their starting locations. For substrate-bound structures, the substrate was removed following Impref minimization. All structures were then aligned to the structure of γ-secretase in complex with APP (PDB 6IYC).

### 4.2. Molecular Docking

Glide High Throughput Virtual Screening (HTVS) and Standard Precision (SP) modes were considered in this study [91,92]. Docking to the inhibitor site (where L685,458, avagacestat, and semagacestat bind) and the modulator site (where E2012 binds) was performed. For γ-secretase structures with small molecules bound at either of the relevant sites, the small molecule was used to define the centroid of the docking grids. Where small molecules were not available to guide grid placement, the docking grid centroid was defined as the centroid of residues found to be within 4.0 Å of L685,458 (to define the γ-secretase inhibitor site) or E2012 (to define the γ-secretase modulator site) in PDB 7D8X (listed in Supplementary Materials Table S7). For both modes of Glide docking, all settings were retained as defaults, with the exception that the Coulomb–van der Waals cutoff limit for pose reporting was raised to +20kcal/mol. For validation of pose prediction, it was additionally specified to report and minimize up to 100 poses per ligand, clustered to an RMSD threshold of 2.0 Å; for docking-based virtual screening, only one pose per ligand was reported (the default) and sampling was adjusted to be either Flexible (the default) or None (Refine Only). Poses were sorted by GlideScore.

### 4.3. Pharmacophore Modelling and Screening

The ePharmacophore feature of Phase [93] was used to generate pharmacophore models against the various γ-secretase structures. For sites with bound ligands, the ligand was used directly to generate the pharmacophore model. For sites without bound ligands, pharmacophore generation was centered at the coordinates of the centroid of the residues listed in Supplementary Materials Table S7. Poses were selected that satisfied at least half of the pharmacophore points and were sorted by PhaseScreenScore [94]. Pharmacophore models derived at the γ-secretase inhibitor site are shown in Supplementary Materials Figure S15, while those derived at the γ-secretase modulator site are shown in Supplementary Materials Figure S16.

### 4.4. Validation of Pose Prediction

The ligands bound to the various γ-secretase structures were used to facilitate validation of pose prediction by the various methods against the various structures. The RMSDs of atomic coordinates of all poses of all ligands obtained by each method against each structure were computed against the bound ligand structures. The RMSD of the top-ranked pose obtained by each method is reported, as well as the lowest RMSD for any pose obtained (referred to as best pose). Docking successes were considered RMSD values below 2.5 Å.

### 4.5. Optimisation of Virtual Screening for Identifying γ-Secretase Inhibitors and γ-Secretase Modulators

To optimise virtual screening for identifying γ-secretase inhibitors and γ-secretase modulators, a library containing known GSMs and relevant decoys was prepared, and a library containing known GSIs and relevant decoys was prepared. Known GSMs and GSIs were obtained from recent comprehensive reviews [28,45], while decoys with similar physicochemical properties to the known molecules were generated using the Database of Useful Decoys: Enhanced (DUD-E) [95]. The GSM library consisted of 78 known GSMs and 6948 decoy molecules, while the GSI library consisted of 23 known GSIs and 1173 decoy molecules. Three-dimensional (3D) structures of all molecules were prepared using LigPrep. One structure per molecule was used; if applicable, only the most likely tautomeric state at physiological pH was retained, and structures featuring the lowest energy ring conformers were retained.

A multi-stage virtual screening procedure was envisioned, wherein virtual screening by one of the faster approaches (Glide HTVS or ePharmacophore screening) was applied, followed by the re-screening of selections of top-ranked ligands by progressively more thorough approaches (Glide SP, followed by Prime MMGBSA). Following this, re-screening of the top-ranked selection of ligands identified using the optimal structure–method combination by Glide SP (considering both fully flexible ligand sampling and refinement only) was performed at all structures. Finally, top ranked ligands selected by the optimal combination of structure with the Glide SP sampling approach were subject to further rescoring by Prime MMGBSA. An implicit membrane was defined using the locations of the transmembrane helices of γ-secretase and was used during the Prime MMGBSA calculations. Optimal cutoffs for selecting ligands for each stage of screening were identified using MCC, as described in “Evaluation of virtual screening performance”.

### 4.6. Evaluation of Virtual Screening Performance

Virtual screening performances were evaluated using receiver operating characteristic (ROC) curves. Performance was assessed in the context of screening by each method at each protein structure/pharmacophore model at each of the inhibitor and modulator sites. Ligands were ranked according to the relevant scoring function for the method. The rates of discovery of hits and decoys were calculated at each point of the ranked list and plotted against one another, with the rate of discovery of hits considered as the true positive rate (TPR; *y*-axis) and the rate of discovery of decoys considered as the false positive rate (FPR; *x*-axis). The area under the curve (AUC) of the plots was determined by applying the trapezoid rule. The Matthews correlation coefficient (*MCC*; (1)) was calculated at each point of the ranked list, assuming the current position on the list as a cutoff for designating true positives (*TP*), false positives (*FP*), true negatives (*TN*), and false negatives (*FN*).

(1) $$MCC=\frac{TP\times TN-FP\times FN}{\sqrt{\left(TP+FP\right)\left(TP+FN\right)\left(TN+FP\right)\left(TN+FN\right)}}\;$$

Optimal cutoffs were reported based on the maximum *MCC* observed within the top 5% to top 25% of the screen. The optimal structure to use with a given approach was designated as the structure yielding the largest *MCC* within the lowest cutoff, i.e., an optimal result would be an *MCC* of 1 (perfect classification; no false positives or false negatives) at 5% of the screen. ROC and *MCC* calculations were conducted using LibreOffice Calc (The Document Foundation, Germany).

### 4.7. Application of Optimised Virtual Screening Procedures to an External Test Set

The ZINC15 [96] Investigational set, which contains a wide range of molecules with known bioactivity at diverse targets, was used to validate the optimized virtual screening procedures. The set was downloaded from ZINC15 and prepared using LigPrep, as described in “Optimization of virtual screening for identifying γ-secretase inhibitors and γ-secretase modulators”. Data on molecule generic naming and molecular targets was sourced (in order of preference) from ZINC15, ChEMBL [97], and PubChem [98]. Shape similarity calculations between the poses of the top remaining ligands and co-complexed γ-secretase inhibitors and modulators were conducted using Shape Screening within the Schrodinger Suite [99]. Calculations were conducted maintaining the molecules in place and using the typed pharmacophore volume scoring approach.

## Figures and Tables

**Table 1:** Virtual screening performance for fast screening approaches for each molecule type ^1^.

	Inhibitors		Modulators	
	Glide HTVS	ePharmacophore	Glide HTVS	ePharmacophore
**6IYC**	0.67 (0.16, 7%)	0.35 (0.06, 15%)	0.65 (0.04, 20%)	0.80 (0.11, 19%)
**6IDF**	0.56 (0.06, 5%)	0.45 (0.03, 5%)	0.64 (0.05, 10%)	0.54 (0.02, 23%)
**6LQG**	0.56 (0.06, 24%)	0.60 (0.12, 12%)	0.51 (0.02, 10%)	0.76 (0.06, 13%)
**6LR4**	0.50 (0.02, 9%)	0.45 (0.11, 12%)	0.58 (0.02, 6%)	0.72 (0.07, 12%)
**7C9I**	0.45 (0.10, 7%)	0.63 (0.17, 6%)	0.56 (0.08, 12%)	0.57 (0.00, 5%)
**7D8X**	0.48 (0.07, 5%)	0.38 (0.07, 5%)	0.63 (0.06, 23%)	0.73 (0.16, 7%)

^1^ For each case, the area under the curve is presented, followed in parentheses by the best Matthews correlation coefficient within the top 5–25% of the screen and the percentage of the screen at which this MCC is observed.

**Table 2:** Performance for enrichment of actives by Glide SP following initial ePharmacophore screening ^1^.

	Inhibitors ^2^		Modulators ^3^	
	SP Flexible	SP Refine Only	SP Flexible	SP Refine Only
**6IYC**	0.99 (0.93, 11%)	0.97 (0.75, 9%)	0.65 (0.19, 15%)	0.54 (0.28, 12%)
**6IDF**	0.95 (0.74, 6%)	0.71 (0.83, 7%)	0.61 (0.15, 10%)	- ^4^
**6LQG**	0.74 (0.27, 23%)	0.77 (0.74, 5%)	0.56 (0.10, 6%)	0.36 (−0.06, 5%)
**6LR4**	0.97 (0.83, 7%)	0.63 (0.74, 5%)	0.50 (0.07, 8%)	0.44 (−0.03, 5%)
**7C9I**	0.80 (0.65, 7%)	0.82 (0.83, 7%)	0.64 (0.26, 10%)	0.35 (0.00, 5%)
**7D8X**	0.94 (0.74, 6%)	0.83 (0.83, 7%)	0.57 (0.00, 17%)	0.70 (0.39, 9%)

^1^ For each case, the area under the curve is presented, followed in parentheses by the best Matthews correlation coefficient within the top 5–25% of the screen and the percentage of the screen at which this MCC is observed. ^2^ The input library for these screens was the top 6% ranked ligands following screening of the initial γ-secretase inhibitor library against the PDB 7C9I-derived ePharmacophore. ^3^ The input library for these screens was the top 7% ranked ligands following screening of the initial γ-secretase modulator library against the PDB 7D8X-derived ePharmacophore. ^4^ No actives returned.

**Table 3:** Performance of Prime MMGBSA for further enrichment of actives.

	Performance ^1^
**Inhibitors ^2^**	0.14 (0.14, 5%)
**Modulators ^3^**	0.81 (0.47, 24%)

^1^ The area under the curve is presented, followed in parentheses by the best Matthews correlation coefficient within the top 5–25% of the screen and the percentage of the screen at which this MCC is observed.^2^ The input library was the top 11% ranked ligands following re-screening at PDB 6IYC by Glide SP with a flexible ligand sampling of the top 6% ranked ligands screened at the PDB 7C9I-derived ePharmacophore.^3^ The input library was the top 9% of ranked ligands following re-screening at PDB 7D8X by Glide SP without flexible ligand sampling (i.e., refinement only) of the top 7% ranked ligands screened at the PDB 7D8X-derived ePharmacophore.

**Table 4:** Molecules identified from screening the ZINC15 investigational set using the γ-secretase inhibitor screening strategy.

ZINC ID	Generic Name	Target Class(es)	Specific Target(s)
ZINC000003919807	AG7088	Protease	Human rhinovirus A protease [50]
ZINC000085548251	A-77003	Protease, lyase	HIV-1 protease [51], carbonic anhydrase II [52]
ZINC000043202141	Oprozomib	Protease	Proteasome subunits beta type 5 and 8 [52]
ZINC000068077856	Foxy-5	Class F GPCR, kinase, co-receptor ^1^	Frizzleds, Ryk, RORs, LRP ^1^ [53,54]
ZINC000082138051	PF-03715455	Kinase	VEGFR1 [54], MAP kinase p38 beta [55], misshapen-like kinase 1 [55]
ZINC000169345692	Peptide T	Surface antigen	CD4 [56]
ZINC000095586643	Crenigacestat	Protease	γ-secretase [57]
ZINC000049694463	Cefcanel daloxate	Transferase ^2^	Penicillin-binding proteins ^2^ [58]
ZINC000090636091	- ^3^	Kinase	G protein-coupled receptor kinases [59]
ZINC000003935423	Droxinavir	Protease	HIV-1 protease [60]
ZINC000027657184	Modipafant	Class A GPCR, voltage-gated ion channel	Platelet-activating factor receptor [61], voltage-dependent L-type calcium channel subunit α_1D_ [61]
ZINC000003830407	Cefazolin	Transferase	Penicillin-binding proteins [62]
ZINC000003917787	- ^3^	Protease	Renin [63], cathepsin D [64]
ZINC000001541366	Ticolubant	Class A GPCR, reductase	Leukotriene B4 receptor 1 [65], arachidonate 5-lipoxygenase [65]
ZINC000002012859	Halofenate	Transcription factor	PPARγ [66]
ZINC000005599165	Doreptide	Class A GPCR ^2^	Dopamine receptors^2^ [67,68]
ZINC000200259560	MK-0767	Transcription factor	PPARα [69], PPARγ [69]
ZINC000003915259	Telinavir	Protease	Human rhinovirus A protease [70], HIV-1 protease [70]
ZINC000206178236	Navarixin	Class A GPCR	CXCR2 [71], CXCR1 [71]
ZINC000118795962	Itacitinib	Kinase	JAK1 [72], JAK2 [72]
ZINC000028257302	- ^3^	Protease	Renin [73]
ZINC000004392972	CP-195543	Class A GPCR	Leukotriene B4 receptor [74]
ZINC000000600399	Lixivaptan	Class A GPCR	Vasopressin receptors (V1a, V2) [75], oxytocin receptor [76]
ZINC000003807687	JTP-4819	Protease	Prolyl endopeptidase [77]
ZINC000003831243	Oxacillin	Transferase	Penicillin-binding protein [78]

^1^ Mimic of Wnt5a; targets listed are possible targets. ^2^ Target assignment based on chemical class. ^3^ Generic name not identifiable in ZINC15, ChEMBL, or PubChem.

**Table 5:** Molecules identified from screening the ZINC15 investigational set using the γ-secretase modulator screening strategy.

ZINC ID	Generic Name	Target Class(es)	Specific Target(s)
ZINC000117704832	PF-04691502	Kinase	AKT [79], PI3Kα [80], mTOR [79,80]
ZINC000003919807	AG7088	Protease	Human rhinovirus A protease [50]
ZINC000034285235	AMG-208	Kinase	MET [81]
ZINC000067172224	E2012	Protease	γ-secretase [82]
ZINC000000005014	Ocinaplon	Protease	SARS-CoV-2 main protease [83]

## Data Availability

The data presented in this study are available on request from the corresponding author.

## Supplementary Materials

The following supporting information can be downloaded at, Table S1: RMSD data for evaluation of Glide HTVS docking for reproducing bound structures of γ-secretase inhibitors and modulators, Table S2: RMSD data for evaluation of Glide SP docking for reproducing bound structures of γ-secretase inhibitors and modulators, Table S3: RMSD data for evaluation of ePharmacophores for reproducing bound structures of γ-secretase inhibitors and modulators, Table S4: Shape similarity of molecules remaining after applying γ-secretase inhibitor screening strategy to ZINC15 Investigational set compared to co-complexed γ-secretase inhibitors, Table S5: Shape similarity of molecules remaining after applying γ-secretase modulator screening strategy to ZINC15 Investigational set compared to E2012, Table S6: Structures of γ-secretase used in this study, Table S7: γ-secretase residues defining the ligand binding sites, Figure S1: Receiver operating characteristic plots and Matthew’s correlation coefficients over the top 5–25% of screens for Glide HTVS screening of library of γ-secretase inhibitors and corresponding decoys from DUD-E, Figure S2: Receiver operating characteristic plots and Matthew’s correlation coefficients over the top 5–25% of screens for ePharmacophore-based screening of library of γ-secretase inhibitors and corresponding decoys from DUD-E, Figure S3: Receiver operating characteristic plots and Matthew’s correlation coefficients over the top 5–25% of screen for Glide HTVS screening of library of γ-secretase modulators and corresponding decoys from DUD-E, Figure S4: Receiver operating characteristic plots and Matthew’s correlation coefficients over the top 5–25% of screens for ePharmacophore-based screening of library of γ-secretase modulators and corresponding decoys from DUD-E, Figure S5: Receiver operating characteristic plots and Matthew’s correlation coefficients over the top 5–25% of screens for Glide SP (with flexible sampling) screening of top 6% of molecules obtained following ePharmacophore-based screening of library containing γ-secretase inhibitors and decoys from DUD-E, Figure S6: Receiver operating characteristic plots and Matthew’s correlation coefficients over the top 5–25% of screens for Glide SP (refine only) screening of top 6% of molecules obtained following ePharmacophore-based screening of library containing γ-secretase inhibitors and decoys from DUD-E, Figure S7: Receiver operating characteristic plots and Matthew’s correlation coefficients over the top 5–25% of screens for Glide SP (with flexible sampling) screening of top 7% of molecules obtained following ePharmacophore-based screening of library containing γ-secretase modulators and decoys from DUD-E, Figure S8: Receiver operating characteristic plots and Matthew’s correlation coefficients over the top 5–25% of screens (second row) for Glide SP screening of top 7% of molecules obtained following ePharmacophore-based screening of library containing γ-secretase modulators and decoys from DUD-E, Figure S9: Receiver operating characteristic plots and Matthew’s correlation coefficients over the top 5–25% of screens for Prime MMGBSA rescreening of Glide SP-based selections derived from the optimally performing structures, Figure S10: 2D structures of the molecules identified following application of the γ-secretase inhibitor screening protocol to the ZINC15 Investigational Set, Figure S11. Ligand interaction diagrams for known γ-secretase inhibitors and molecules selected from the ZINC15 Investigational set with at least limited shape similarity (>0.200) to these, Figure S12: 2D structures of the molecules identified following application of the γ-secretase modulator screening protocol to the ZINC15 Investigational Set, Figure S13: Ligand interaction diagrams for E2012 as posed against the 7D8X ePharmacophore in the validation of approaches for reproducing bound ligand structure to γ-secretase, E2012 as posed following application of the γ-secretase modulator screening protocol, and ocinaplon, Figure S14: Distributions of targets reported for molecules in the ZINC Investigational set, the molecules remaining from the ZINC Investigational set following application of the inhibitor screening strategy, and the molecules remaining from the ZINC Investigational set following application of the modulator screening strategy, Figure S15: ePharmacophores generated at inhibitor-binding site of each γ-secretase structure, Figure S16: ePharmacophores generated at modulator-binding site of each γ-secretase structure.
