# Supplementary material for: Evaluation of Virtual Screening Strategies for the Identification of γ-Secretase Inhibitors and Modulators
Source: Molecules. 2021 Dec 28;27(1):176. doi: 10.3390/molecules27010176 (PMC8746326; doi:10.3390/molecules27010176)
Supplement: Supplementary file 1 [file molecules-27-00176-s001.zip › molecules-1524050 supplementary.pdf]

molecules

MDPI

Supplementary Materials

Evaluation of Virtual Screening Strategies for the Identification of γ-Secretase Inhibitors and Modulators

Alicia Ioppolo [1], Melissa Eccles [1], David Groth [1], Giuseppe Verdile [2], Mark Agostino [1,3,*]

[1] Curtin Health and Innovation Research Institute, Curtin Medical School, Curtin University, Bentley WA 6102, Australia; alicia.ioppolo@postgrad.curtin.edu.au (A.I.); melissa.eccles@postgrad.curtin.edu.au (M.E.); D.Groth@curtin.edu.au (D.G.)
[2] School of Medical and Health Sciences, Edith Cowan University, Joondalup, WA 6027, Australia; giuseppe.verdile@curtin.edu.au
[3] Curtin Institute for Computation, Curtin University, Bentley WA 6102, Australia
* Correspondence: mark.agostino@curtin.edu.au

Table S1. Evaluation of Glide HTVS docking for reproducing bound structures of γ-secretase inhibitors and modulators.[a.]

	E2012	Avagacestat	Semagacestat	L685,458	Means
6IYC	10.4, 3.6 (4)	9.4, 9.4 (1)	5.2, 3.9 (6)	2.9, 2.9 (1) [b]	7.0, 5.0
6IDF	9.9, 6.5 (3)	6.1, 5.8 (2)	5.0, 4.7 (5)	- [c]	7.0, 5.7
6LQG	10.6, 5.3 (4)	6.8, 5.2 (2)	5.1, 5.1 (1)	- [c]	7.5, 5.2
6LR4	8.0, 8.0 (1)	- [c]	0.6, 0.6 (1)	- [c]	4.3, 4.3
7C9I	10.3, 3.5 (5)	- [c]	6.3, 6.3 (1)	- [c]	8.3, 4.9
7D8X	3.5, 3.5 (1)	9.6, 9.6 (1)	7.8, 7.8 (1)	- [c]	7.0, 7.0

[a]Values reported are RMSD of top-ranked pose, RMSD of best fitting pose and rank of the best fitting pose (in parentheses). All values reported in angstroms. [b]RMSD computed against L685,458 in PDB 7D8X. [c]No poses obtained for this ligand against this model.

Table S2. Evaluation of Glide SP docking for reproducing bound structures of γ-secretase inhibitors and modulators.[a.]

PDB ID	E2012	Avagacestat	Semagacestat	L685,458	Mean values
6IYC	10.4, 2.2 (21)	2.1, 2.1 (1)	1.4, 1.2 (2)	8.9, 2.3 (12) [b]	5.7, 2.0
6IDF	9.4, 5.5 (35)	1.9, 1.9 (1)	1.0, 1.0 (1)	8.1, 6.7 (6) [b]	5.1, 3.8
6LQG	7.8, 2.5 (38)	1.9, 1.9 (1)	1.9, 1.9 (1)	5.0, 5.0 (1) [b]	4.2, 2.8
6LR4	9.9, 3.6 (41)	7.3, 7.3 (1)	1.0, 0.8 (3)	8.7, 7.1 (2) [b]	6.7, 4.7
7C9I	10.2, 2.7 (23)	2.8, 2.8 (1)	1.5, 1.5 (1)	11.2, 4.6 (6)	6.4, 2.9
7D8X	10.0, 2.0 (36)	9.2, 2.6 (2)	1.3, 1.3 (1)	4.0, 4.0 (1)	6.1, 2.5

[a]Values reported are RMSD of top-ranked pose, RMSD of best fitting pose and rank of the best fitting pose (in parentheses). All values reported in angstroms. [b]RMSD computed against L685,458 in PDB 7D8X.

Table S3. Evaluation of ePharmacophores for reproducing bound structures of γ-secretase inhibitors and modulators.[a.]

	E2012	Avagacestat	Semagacestat	L685,458	Means
6IYC	9.5, 8.9 (5)	7.5, 7.5 (2)	8.6, 8.6 (1)	10.1, 4.1 (6) [b]	8.9, 7.3
6IDF	- [c]	8.6, 3.3 (26)	4.8, 4.8 (1)	10.0, 8.4 (4) [b]	7.8, 5.5
6LQG	- [c]	3.1, 2.9 (3)	3.8, 3.8 (1)	10.7, 5.1 (2) [b]	5.9, 3.9
6LR4	10.0, 9.3 (17)	5.6, 5.6 (2)	1.6, 1.6 (1)	- [c]	5.7, 5.5
7C9I	10.6, 10.5 (21)	8.1, 3.3 (23)	1.5, 1.5 (1)	9.8, 7.6 (3)	7.5, 5.7
7D8X	1.9, 1.9 (1)	8.4, 8.2 (2)	1.7, 1.5 (2)	5.1, 5.1 (1)	4.3, 4.2

[a]Values reported are RMSD of top-ranked pose, RMSD of best fitting pose and rank of the best fitting pose (in parentheses). All values reported in angstroms. [b]RMSD computed against L685,458 in PDB 7D8X. [c]No poses obtained for this ligand against this model.

Table S4. Shape similarity of molecules remaining after applying γ-secretase inhibitor screening strategy to ZINC15 Investigational set compared to co-complexed γ-secretase inhibitors.

		Similarity to		
**ZINC ID**	**Generic name (if available)**	**Avagacestat**	**Semagacestat**	**L685,458**
ZINC000003919807	AG7088	0.118	0.187	0.095
ZINC000085548251	A-77003	0.148	0.159	0.128
ZINC000043202141	Oprozomib	0.165	**0.410**	0.150
ZINC000068077856	Foxy-5	0.108	**0.216**	0.189
ZINC000082138051	PF-03715455	0.100	0.059	0.130
ZINC000169345692	Peptide T	0.090	0.047	0.049
ZINC000095586643	Crenigacestat	0.123	**0.605**	0.134
ZINC000049694463	Cefcanel daloxate	0.069	0.074	0.141
ZINC000090636091	-	0.071	0.198	0.053
ZINC000003935423	Droxinavir	0.135	**0.302**	**0.216**
ZINC000027657184	Modipafant	0.072	0.123	0.100
ZINC000003830407	Cefazolin	0.103	0.151	0.053
ZINC000003917787	-	0.108	0.186	0.170
ZINC000001541366	Ticolubant	0.078	0.037	0.051
ZINC000002012859	Halofenate	0.047	0.145	0.111
ZINC000005599165	Doreptide	0.088	0.186	0.062
ZINC000200259560	MK-0767	0.009	0.028	0.094
ZINC000003915259	Telinavir	0.132	0.180	0.168
ZINC000206178236	Navarixin	0.085	0.117	0.050
ZINC000118795962	Itacitinib	0.115	0.052	0.102
ZINC000028257302	-	0.112	0.043	0.122
ZINC000004392972	CP-195543	0.075	0.136	0.072
ZINC000000600399	Lixivaptan	0.116	0.175	0.052
ZINC000003807687	JTP-4819	0.124	0.145	0.067
ZINC000003831243	Oxacillin	**0.200**	**0.264**	0.077

Table S5. Shape similarity of molecules remaining after applying γ-secretase modulator screening strategy to ZINC15 Investigational set compared to E2012.

**ZINC ID**	**Generic Name (If Available)**	**Similarity to E2012**
ZINC000117704832	PF-04691502	0.118
ZINC000003919807	AG7088	0.104
ZINC000034285235	AMG-208	0.176
ZINC000067172224	E2012	0.317
ZINC000000005014	Ocinaplon	0.244

Table S6. Structures of γ-secretase used in this study.

PDB ID	Substrate	Inhibitor	Modulator	Resolution (Å)
6IYC	APP	-	-	2.6
6IDF	Notch	-	-	2.7
6LQG	-	Avagacestat	-	3.1
6LR4	-	Semagacestat	-	3.0
7C9I	-	L685,458	-	3.1
7D8X	-	L685,458	E2012	2.6

Table S7. γ-secretase residues defining the ligand binding sites.

Inhibitor Site		Modulator Site	
Presenilin N-terminal fragment	Presenilin C-terminal fragment	Presenilin N-terminal fragment	Nicastrin
Tyr77	Val379	Phe105	Ile242
Leu85	Lys380	Tyr106	Asn243
Ile143	Leu381	Phe177	
Thr147	Gly382	Ile180	
Leu150	Leu383	Val236	
Tyr256	Gly384	Tyr240	
Asp257	Asp385		
Val261	Phe388		
Leu268	Leu418		
Leu271	Thr421		
Val272	Leu422		
Gln276	Leu425		
Leu282	Lys430		
Leu286	Ala431		
Ile287	Leu432		
	Pro433		
	Ala434		
	Leu435		

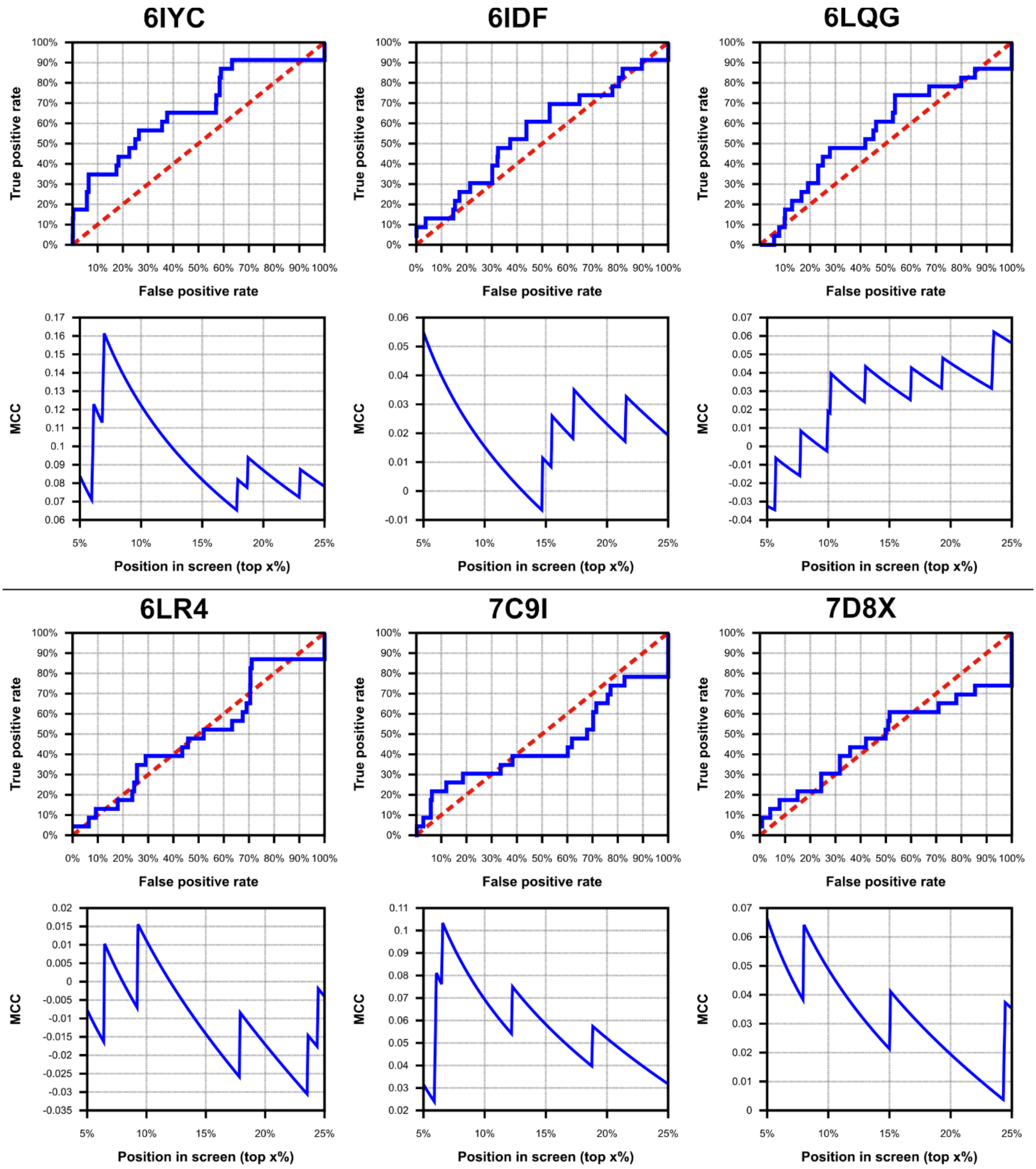

Figure S1. Receiver operating characteristic plots (first row; random performance indicated by red dashed line) and Matthew's correlation coefficients over the top 5-25% of screens (second row) for Glide HTVS screening of library of γ-secretase inhibitors and corresponding decoys from DUD-E.

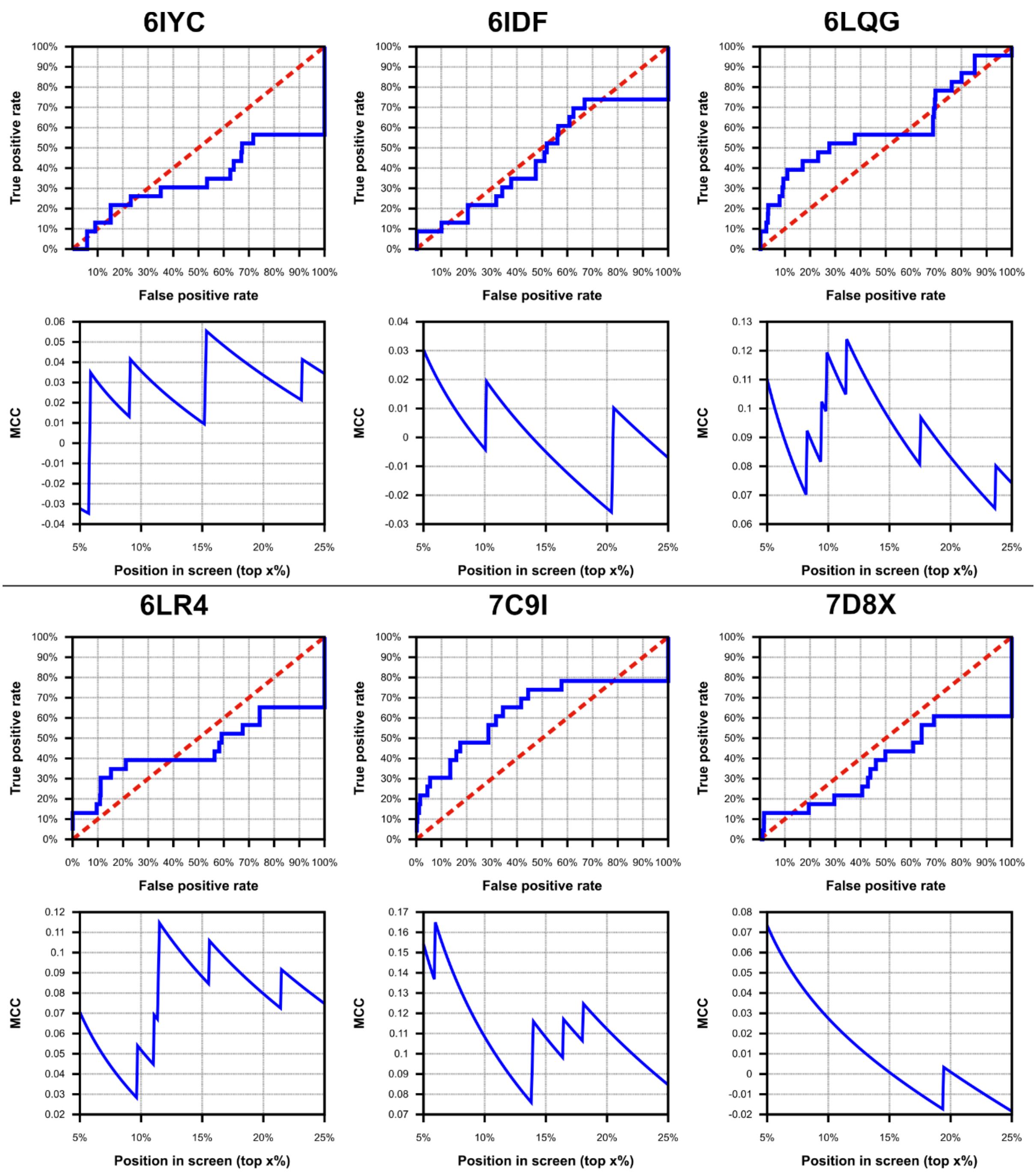

Figure S2. Receiver operating characteristic plots (first row; random performance indicated by red dashed line) and Matthew's correlation coefficients over the top 5-25% of screens (second row) for ePharmacophore-based screening of library of γ-secretase inhibitors and corresponding decoys from DUD-E.

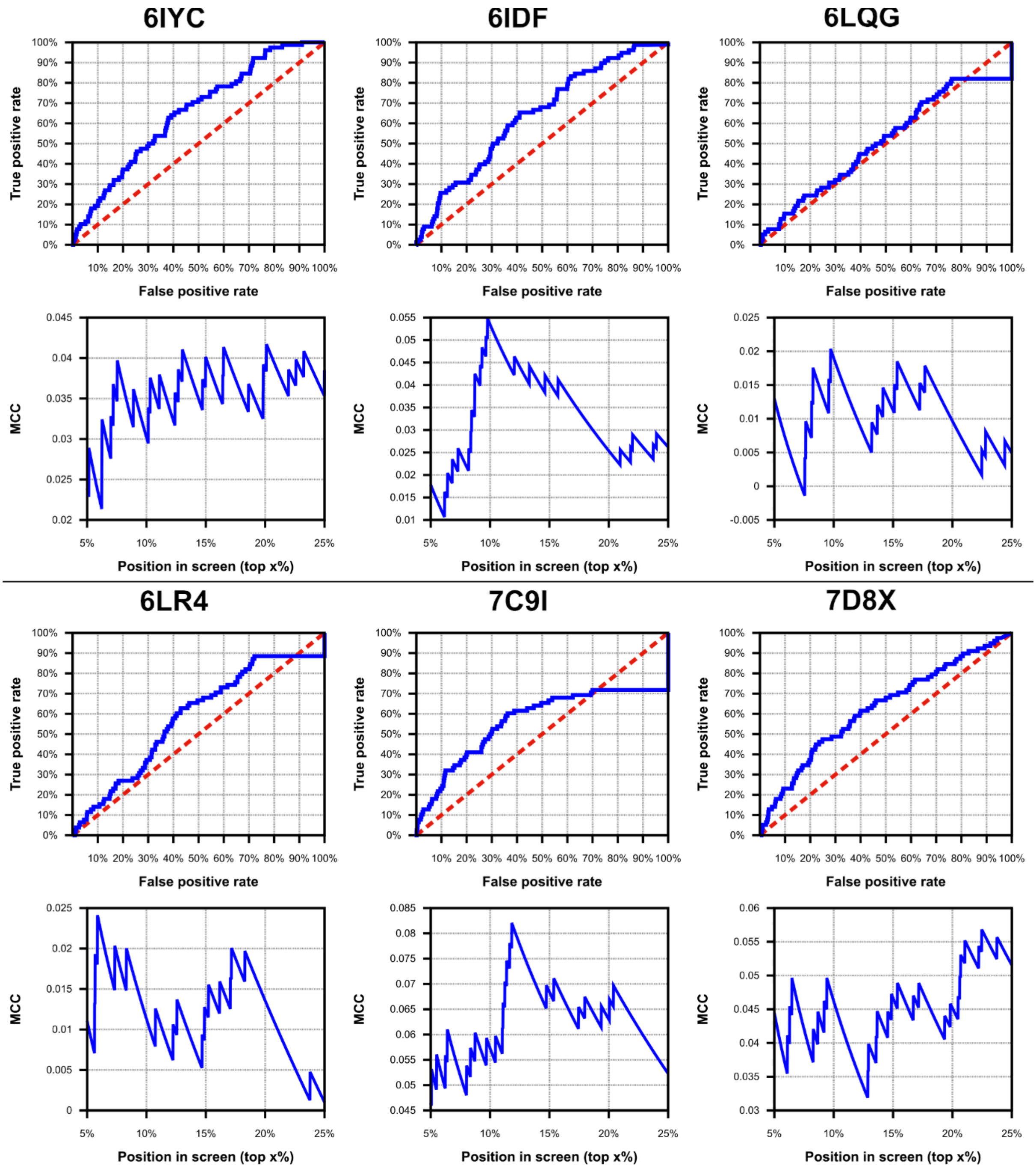

Figure S3. Receiver operating characteristic plots (first row; random performance indicated by red dashed line) and Matthew's correlation coefficients over the top 5-25% of screens (second row) for Glide HTVS screening of library of γ-secretase modulators and corresponding decoys from DUD-E.

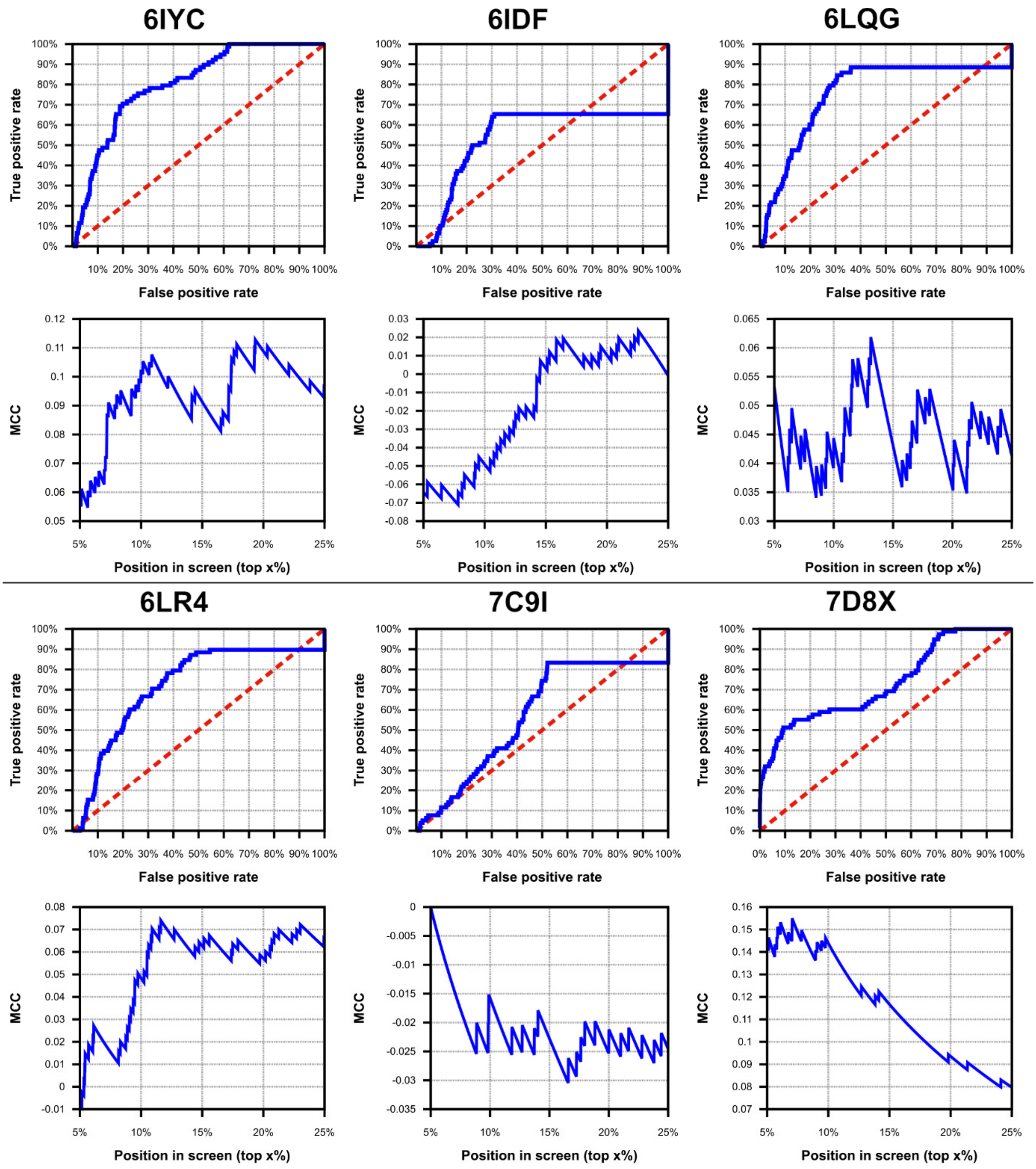

Figure S4. Receiver operating characteristic plots (first row; random performance indicated by red dashed line) and Matthew's correlation coefficients over the top 5-25% of screens (second row) for ePharmacophore-based screening of library of γ-secretase modulators and corresponding decoys from DUD-E.

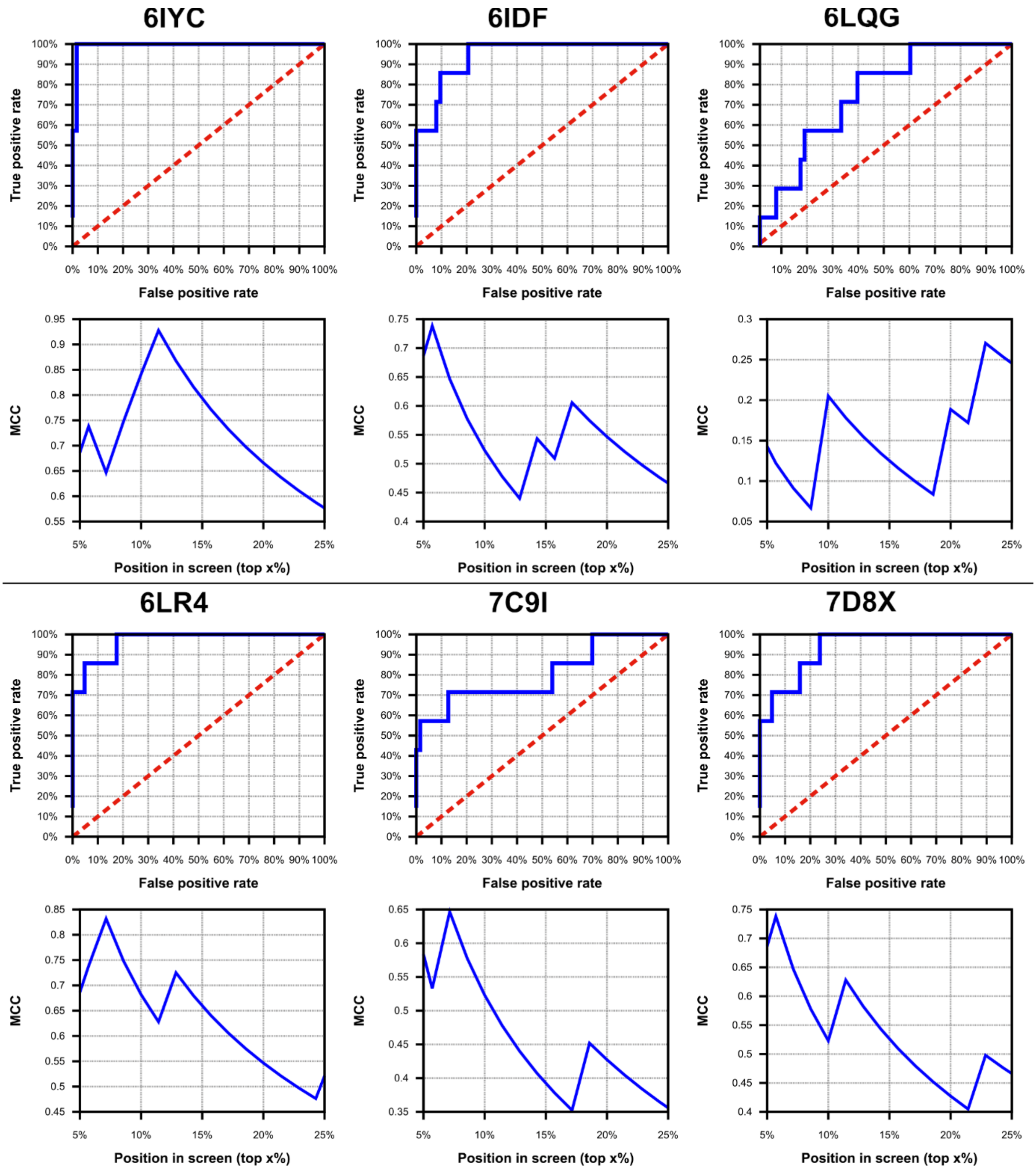

Figure S5. Receiver operating characteristic plots (first row; random performance indicated by red dashed line) and Matthew's correlation coefficients over the top 5-25% of screens (second row) for Glide SP (with flexible sampling) screening of top 6% of molecules obtained following ePharmacophore-based screening of library containing γ-secretase inhibitors and decoys from DUD-E.

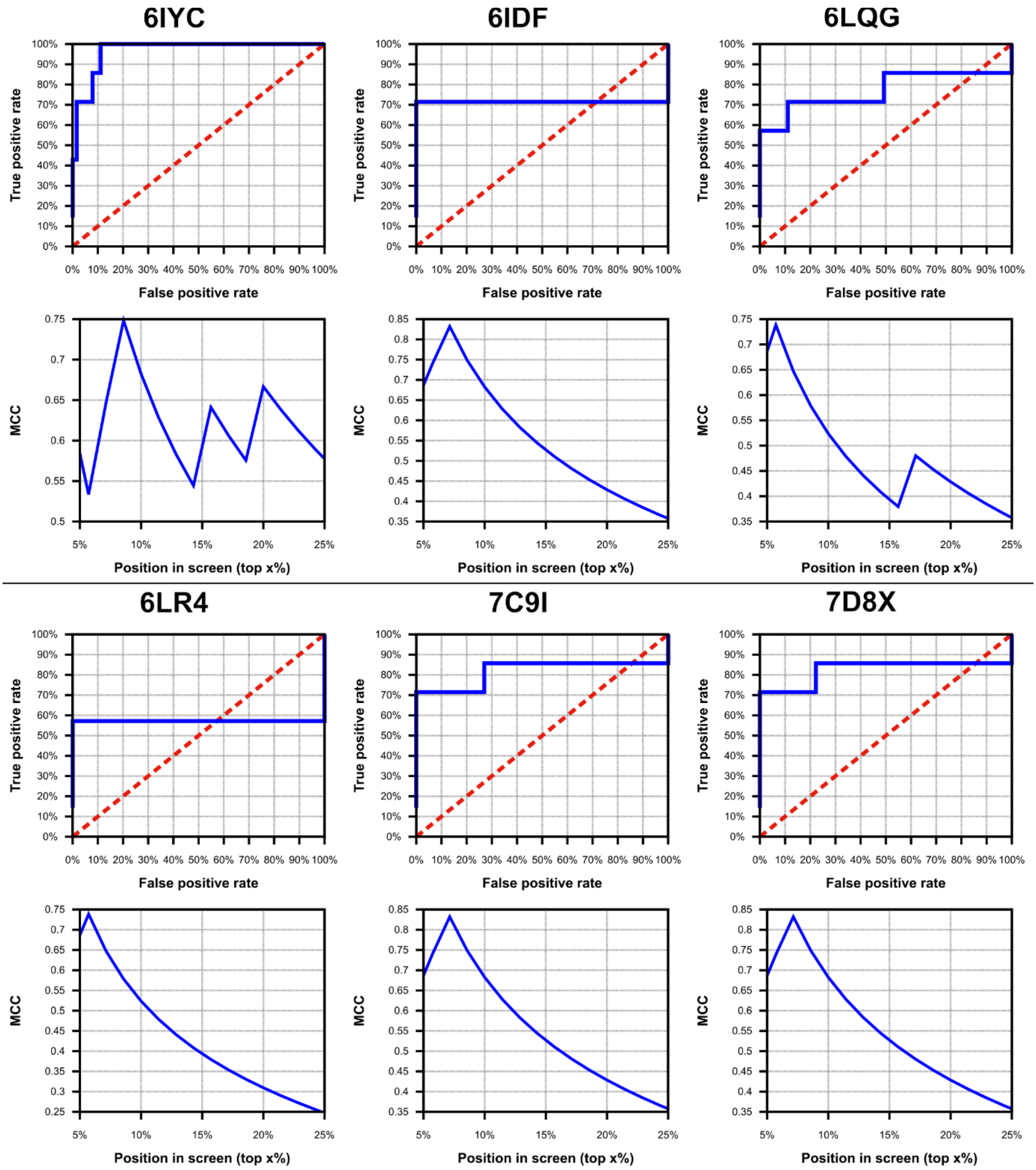

Figure S6. Receiver operating characteristic plots (first row; random performance indicated by red dashed line) and Matthew's correlation coefficients over the top 5-25% of screens (second row) for Glide SP (refine only) screening of top 6% of molecules obtained following ePharmacophore-based screening of library containing γ-secretase inhibitors and decoys from DUD-E.

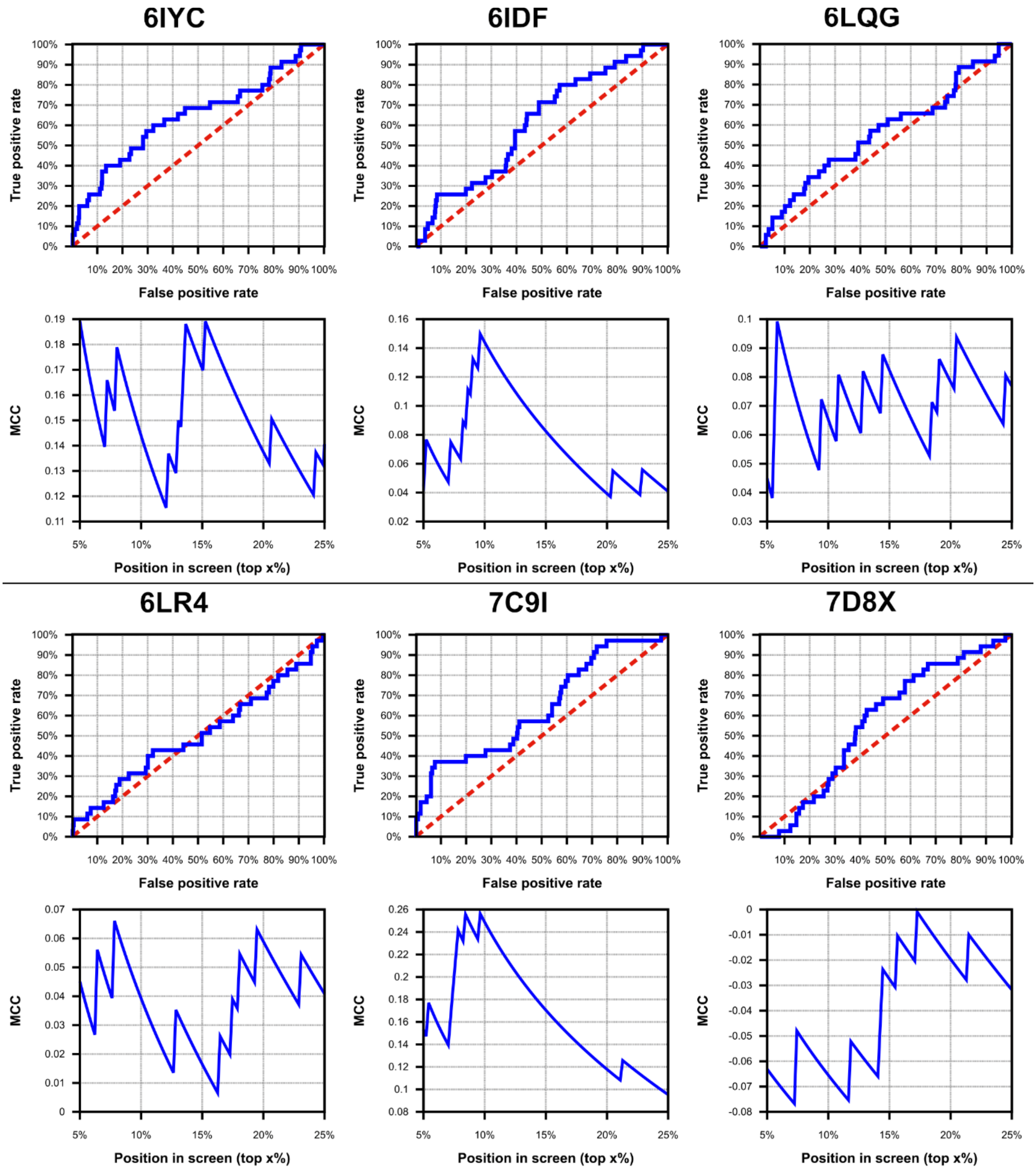

Figure S7. Receiver operating characteristic plots (first row; random performance indicated by red dashed line) and Matthew's correlation coefficients over the top 5-25% of screens (second row) for Glide SP (with flexible sampling) screening of top 7% of molecules obtained following ePharmacophore-based screening of library containing γ-secretase modulators and decoys from DUD-E.

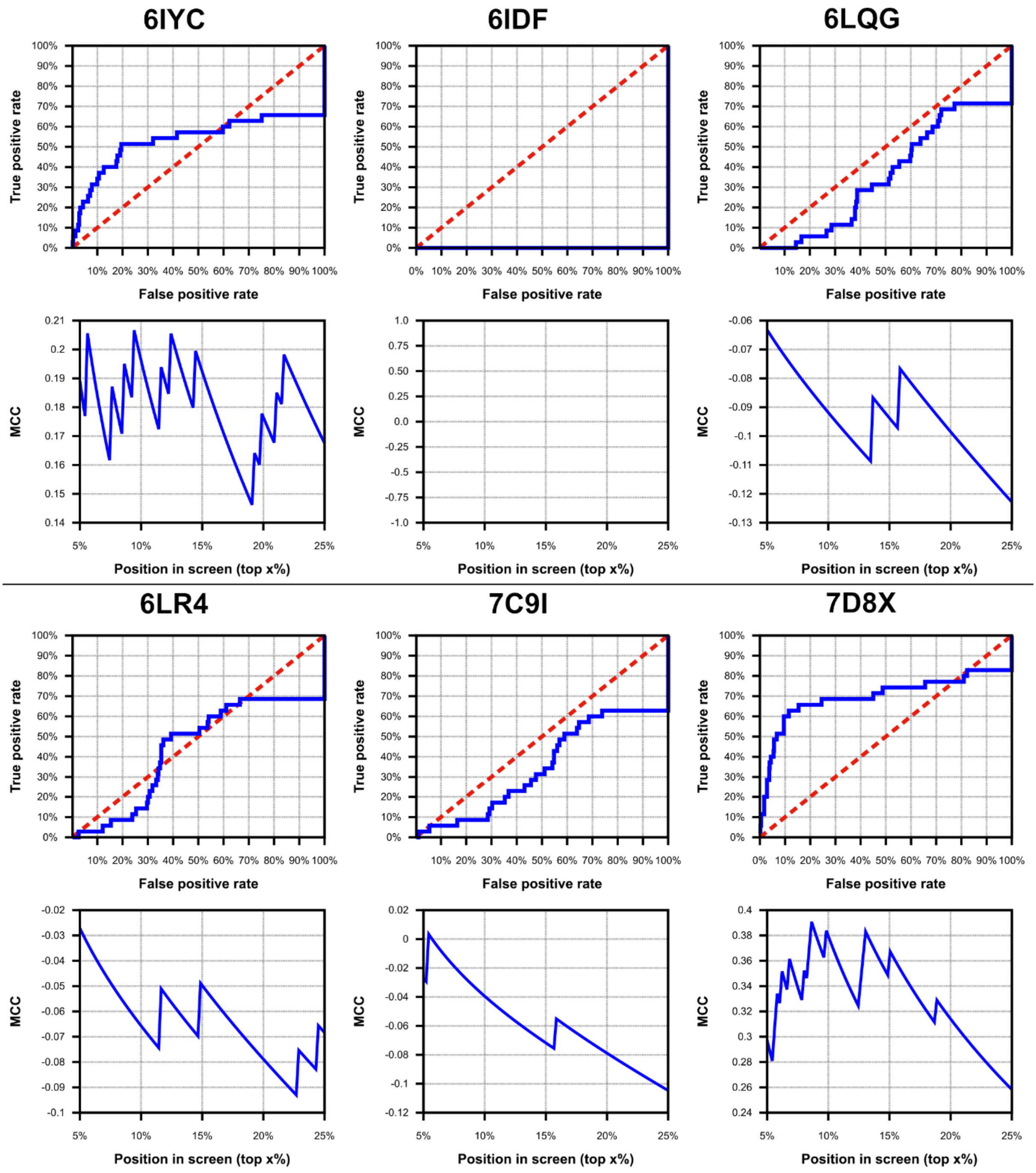

Figure S8. Receiver operating characteristic plots (first row; random performance indicated by red dashed line) and Matthew's correlation coefficients over the top 5-25% of screens (second row) for Glide SP (refine only) screening of top 7% of molecules obtained following ePharmacophore-based screening of library containing γ-secretase modulators and decoys from DUD-E.

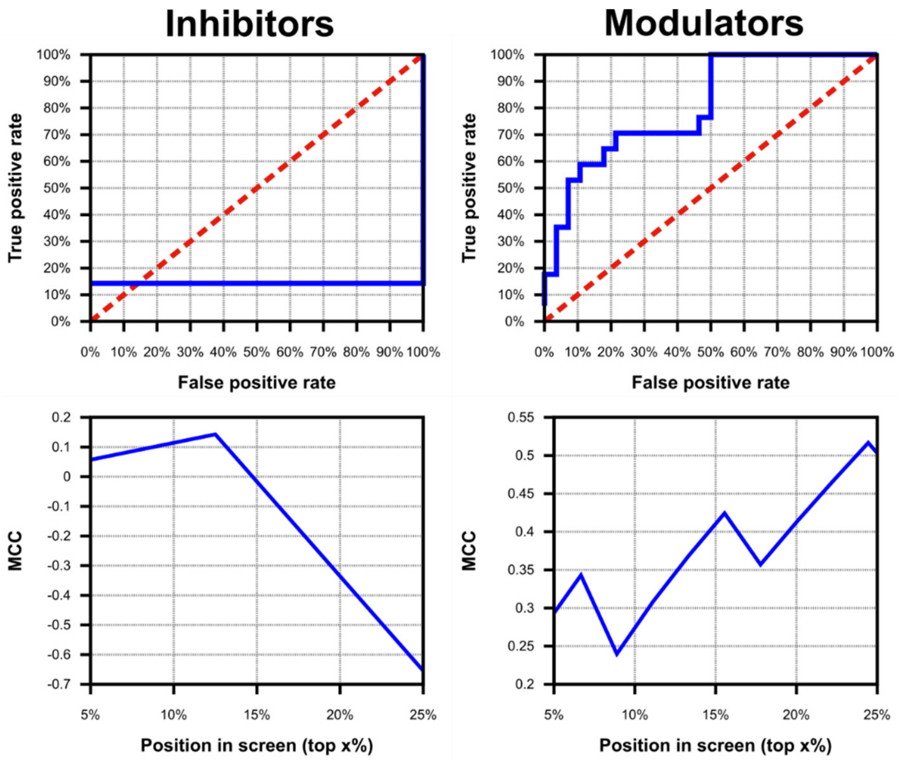

Figure S9. Receiver operating characteristic plots (first row; random performance indicated by red dashed line) and Matthew's correlation coefficients over the top 5-25% of screens (second row) for Prime MMGBSA rescreening of Glide SP-based selections derived from the optimally performing structures.

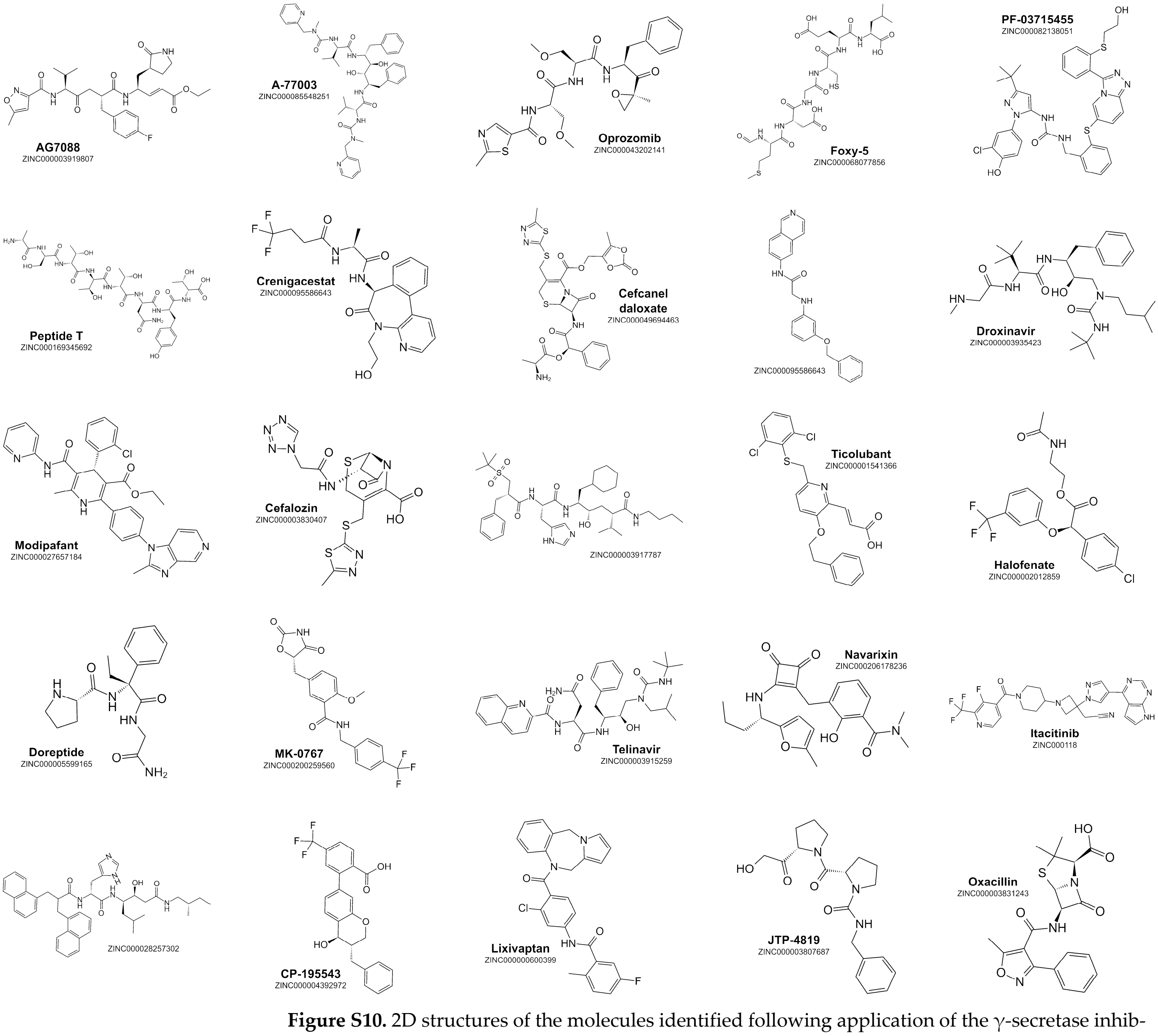

Figure S10. 2D structures of the molecules identified following application of the γ-secretase inhibitor screening protocol to the ZINC15 Investigational Set.

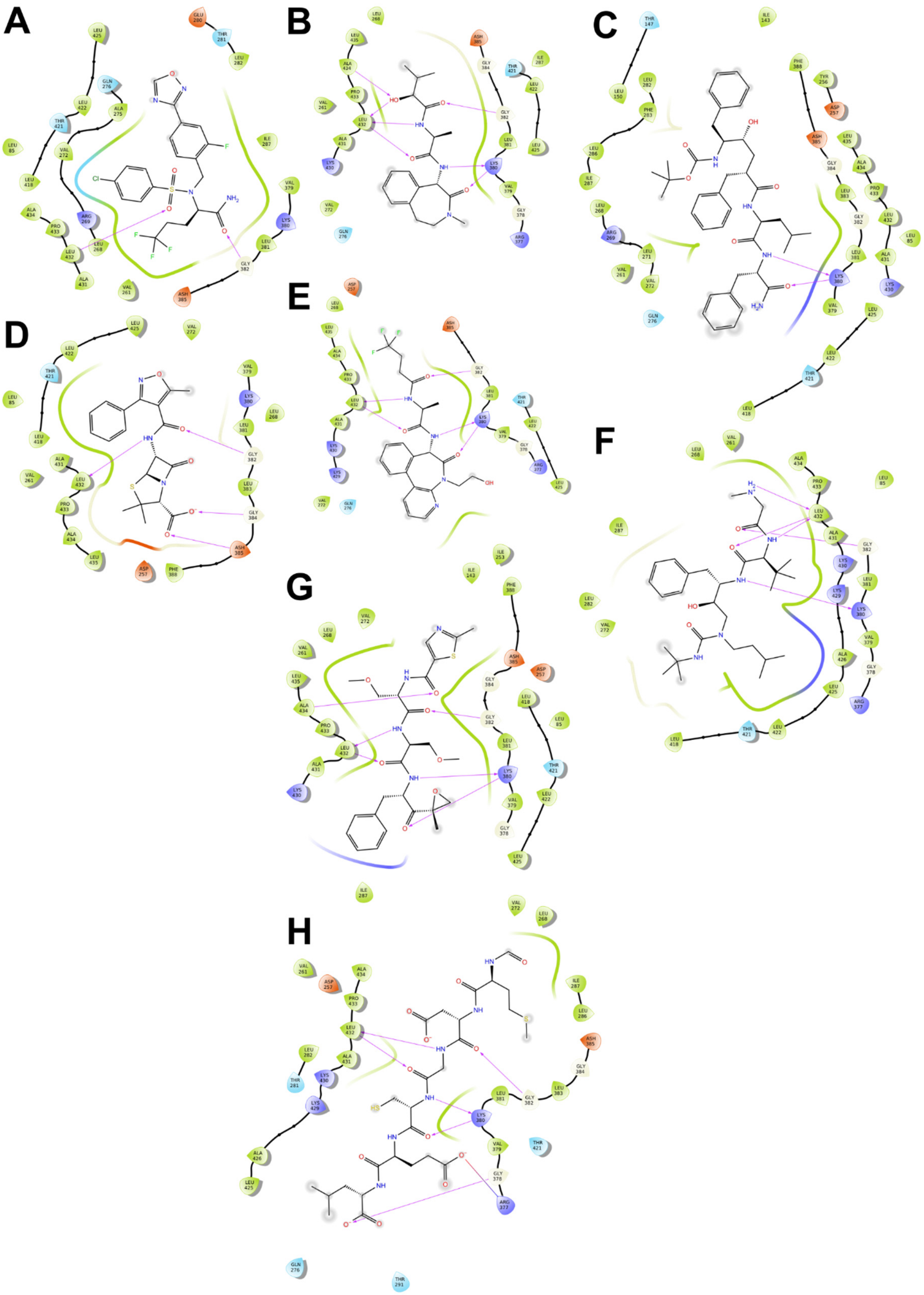

Figure S11. Ligand interaction diagrams for known γ-secretase inhibitors (A-C) and molecules selected from the ZINC15 Investigational set with at least limited shape similarity (>0.200) to these (D-H). A. Avagacestat, B. Semagacestat. C. L685,458. D. Oxacillin. E. Crenigacestat (known γ-secretase inhibitor). F. Droxinavir. G. Oprozomib. H. Foxy-5. Protein residues within 4.0 Å of the ligand are shown. Legend: pink arrows–hydrogen bonds; grey halos–solvent accessibility of ligand atoms; thick coloured lines–protein surface near ligand.

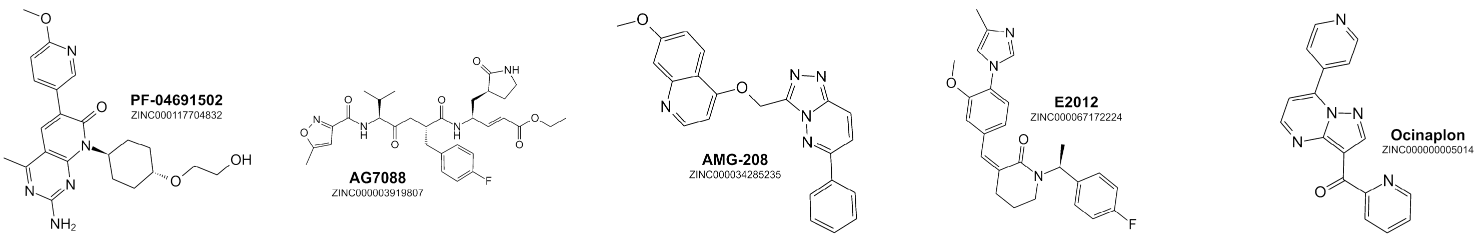

Figure S12. 2D structures of the molecules identified following application of the γ-secretase modulator screening protocol to the ZINC15 Investigational Set.

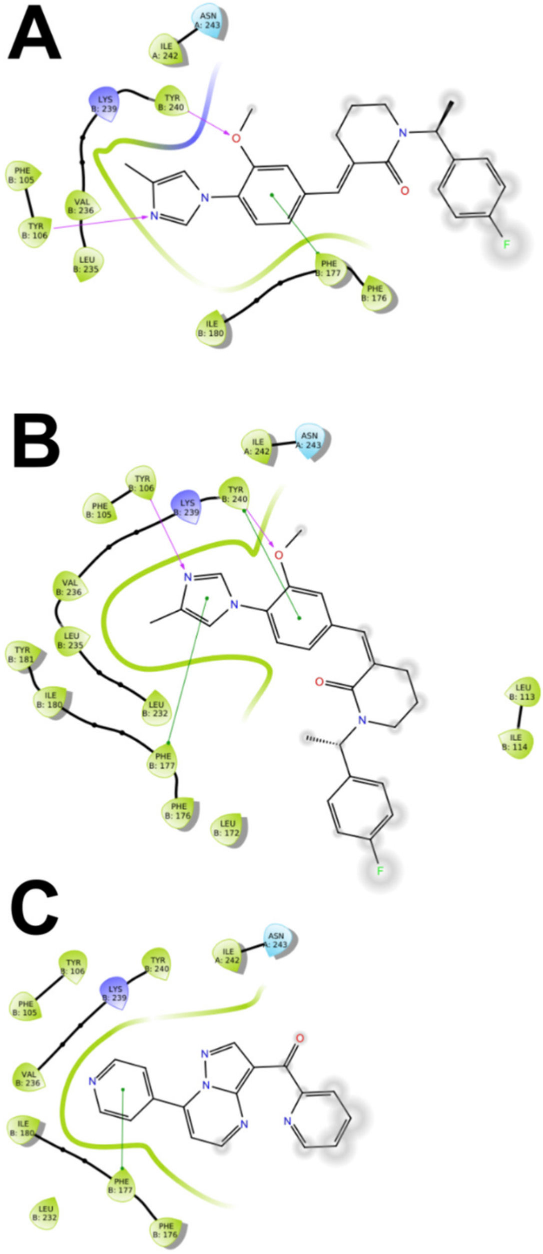

Figure S13. Ligand interaction diagrams for E2012 as posed against the 7D8X ePharmacophore in the validation of approaches for reproducing bound ligand structure to γ-secretase (A), E2012 as posed following application of the γ-secretase modulator screening protocol (B), and ocinaplon (C). Protein residues within 4.0 Å of the ligand are shown. Chain A denotes nicastrin and chain B denotes presenilin. Legend: pink arrows–hydrogen bonds; green lines–π-π interactions; grey halos–solvent accessibility of ligand atoms; thick coloured lines–protein surface near ligand.

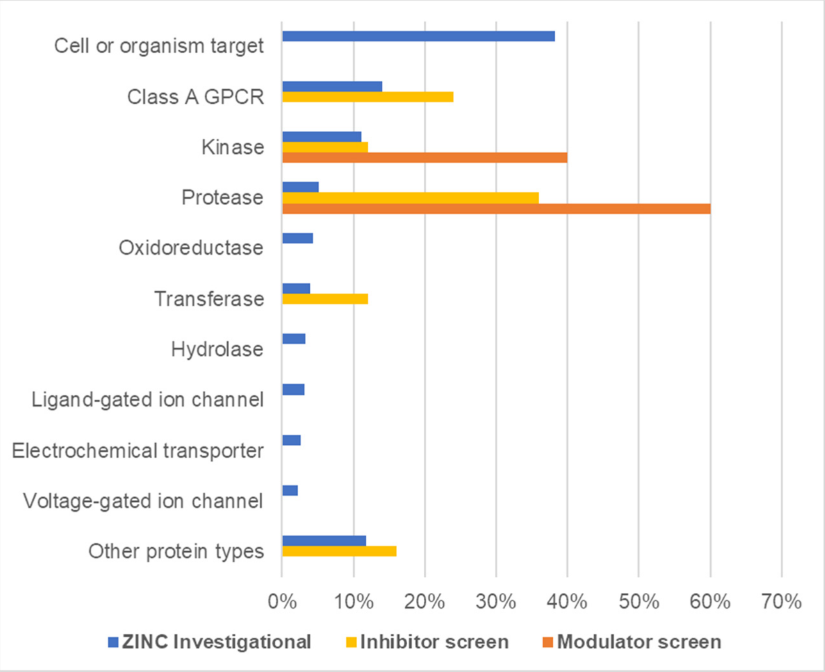

Figure S14. Distributions of targets reported for molecules in the ZINC Investigational set (blue bars), the molecules remaining from the ZINC Investigational set following application of the inhibitor screening strategy (yellow bars), and the molecules remaining from the ZINC Investigational set following application of the modulator screening strategy (red bars).

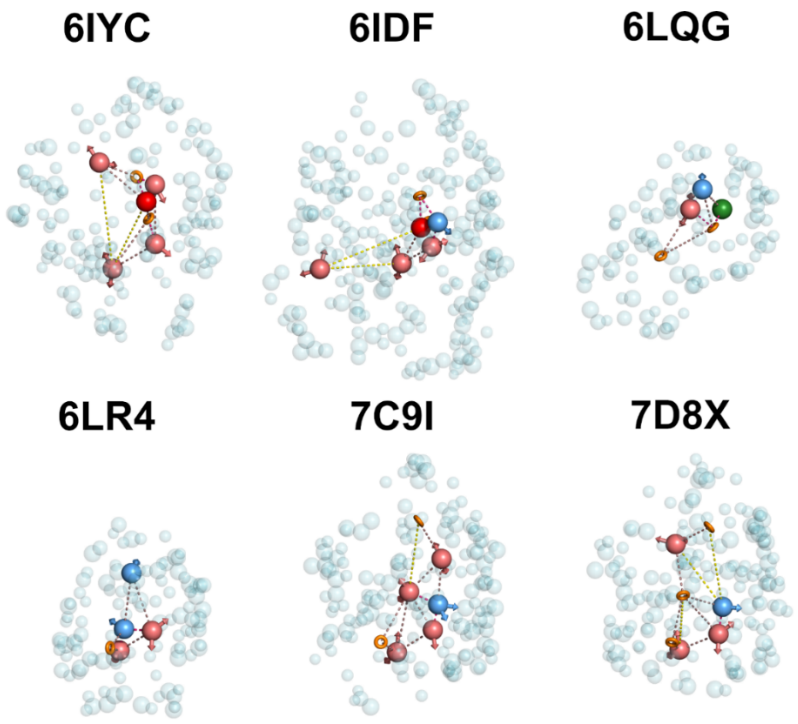

Figure S15. ePharmacophores generated at inhibitor-binding site of each γ-secretase structure. Legend: pink spheres–hydrogen bond acceptor site; red spheres–negatively charged site; blue spheres –hydrogen bond donor site; green spheres–hydrophobic site; orange rings–aromatic site; blue transparent spheres–excluded volumes; yellow dashes–site-to-site distance greater than 8.0 Å; violet dashes–site-to-site distance between 4.0 and 8.0 Å; pink dashes–site-to-site distance less than 4.0 Å.

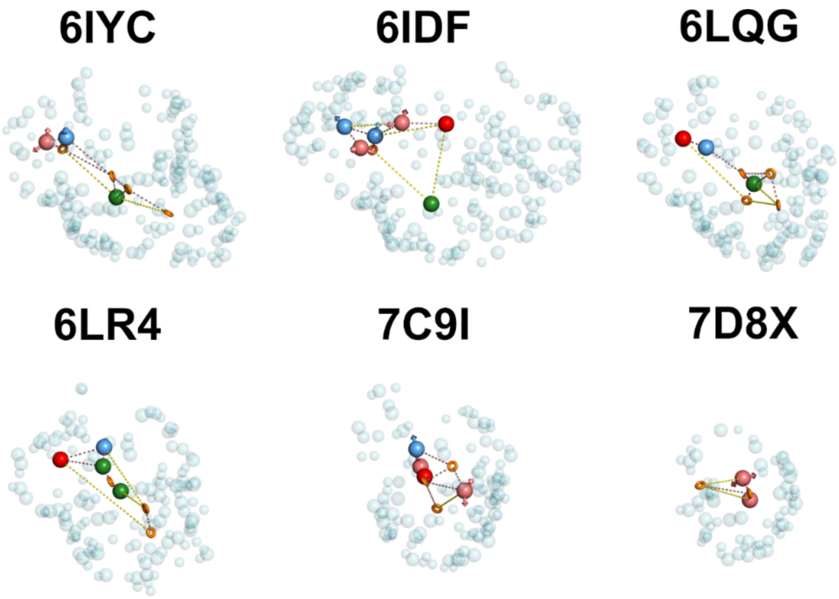

Figure S16. ePharmacophores generated at modulator-binding site of each γ-secretase structure. Legend: pink spheres–hydrogen bond acceptor site; red spheres–negatively charged site; blue spheres–hydrogen bond donor site; green spheres–hydrophobic site; orange rings–aromatic site; blue transparent spheres–excluded volumes; yellow dashes–site-to-site distance greater than 8.0 Å; violet dashes–site-to-site distance between 4.0 and 8.0 Å; pink dashes–site-to-site distance less than 4.0 Å.
